# Polymethoxyflavones extracted from *Bauhinia championii* alleviate LPS-induced acute lung injury by ameliorating endoplasmic reticulum stress

**DOI:** 10.3389/fphar.2025.1544916

**Published:** 2025-06-11

**Authors:** Yuanyuan Li, Xiaolan Yang, Wenjing Ye, Junsong Jing, Ranran Chen, Lianhao Wu, Zhenqiang You, Sheng Zhang, Jing Shi

**Affiliations:** ^1^ School of Pharmaceutical Sciences, Hangzhou Medical College, Hangzhou, China; ^2^ School of Public Health, Hangzhou Medical College, Hangzhou, China; ^3^ Center for Safety Evaluation and Research, Hangzhou Medical College, Hangzhou, China

**Keywords:** polymethoxyflavones, *Bauhinia championii*, acute lung injury, endoplasmic reticulum stress, network analysis

## Abstract

**Background:**

Acute lung injury (ALI), a critical respiratory condition, often escalates into acute respiratory distress syndrome, which is associated with significant morbidity and mortality. *Bauhinia championii*, a botanical drug used in traditional Chinese medicine, is reputed for its antioxidative and anti-hypoxia effects. However, the active metabolites within *B. championii* and their mechanisms of action in alleviating ALI remain to be elucidated.

**Methods:**

A comprehensive literature review and database search within Chemistry Database were conducted to compile a complete profile of the metabolites identified in *B. championii*. Utilizing network analysis, we predicted potential targets of metabolites in *B. championii* (MBC) for ALI treatment. A protein-protein interaction (PPI) network was constructed using Cytoscape 3. 9. 1, complemented by GO annotations and KEGG pathway enrichment analyses via the DAVID online platform. The isolation and characterization of polymethoxyflavones (PMFs) from *B. championii* were performed using HPLC and confirmed by LC-MS. *In vivo* pharmacological assessments were executed to substantiate the network analysis predictions. Moreover, the Autodock software facilitated molecular docking studies to elucidate the role of endoplasmic reticulum (ER) stress modulation in ALI treatment by PMFs.

**Results:**

17 known MBC were identified in which 7 active metabolites of flavonoids were used as predictive targets. 122 target genes associated with both MBC and ALI were tested for KEGG and GO enrichment analyses, which indicated these target genes involvement in antioxidant, anti-inflammatory, and anti-apoptotic pathways. The PMFs were extracted from *B. championii* and identified as 5, 6, 7, 3′, 4′-pentamethoxyflavone, 5, 6, 7, 3′, 4′, 5′-hexamethoxyflavone, 5, 7, 3′, 4′, 5′-pentamethoxyflavone, 5, 6, 7, 5′-tetramethoxy-3′, 4′-methylenedioxyflavone and 5, 7, 5′-trimethoxy-3′, 4′-methylenedioxyflavone. PMFs were effective in alleviating LPS-induced pulmonary inflammatory responses for releasing ALI. In addition, PMFs inhibited the secretion of GSH-Px and CAT, reduced the accumulation of HYP and MDA as well as the infiltration of inflammatory cells, not to mention alleviated LPS-induced apoptosis by inhibiting the Caspase 3-mediated apoptosis pathway. Furthermore, the PMFs can spontaneously bind to multiple ER stress targets to exert the effect of calming ER stress to alleviate ALI.

**Conclusion:**

PMFs inhibited the expression of inflammatory cytokines and reduced oxidative stress injury to resist apoptosis in lung. Moreover, PMFs attenuated LPS-induced ER stress activation by regulating ER stress related targets, which in turn alleviated ALI.

## 1 Introduction

Acute lung injury (ALI) manifests as an acute hypoxic respiratory insufficiency, precipitated by damage to alveolar epithelial cells and capillary endothelial cells from a spectrum of direct and indirect insults. This damage leads to widespread interstitial and alveolar edema. In its severe form, ALI can progress to acute respiratory distress syndrome (ARDS), with an incidence rate approaching 40% and associated with a mortality rate of 40%–50% (Wang et al., 2020). In addition to increased mortality during and after hospital discharge, patients who survive ARDS are likely to have serious long-term sequelae (Shaw et al., 2019). Currently, available therapies for ALI/ARDS are categorized into supportive therapies and pharmacologic interventions. The lung protection strategy of mechanical ventilation is presently considered the only supportive therapy that effectively improves survival (Luh and Chiang, 2007). Physiologically-based pharmacotherapy is the use of medicine that affects ventilation, and diffusion or perfusion is used to alleviate the symptoms of ALI. In fact, there is no effective pharmacologic therapy for ALI/ARDS that substantially reduces mortality and improves patient quality of life (Fan et al., 2018; Ortiz-Diaz et al., 2013).

It is well known that ALI/ARDS is an inflammatory disease of the lungs. The inflammatory response is widely considered an essential feature contributing to a variety of lung diseases (Lee et al., 2021). The release of inflammatory signals causes lung injury characterized by inflammatory cell infiltration, which induces apoptosis of APCs involved in the early pathological process of ALI (Martin et al., 2005). Severe pneumonia increases the production of pro-inflammatory cytokines such as IL-6, Il-1β, TNF-α and IFN-γ, which attract neutrophils and increase the production of reactive oxygen species (ROS) to exacerbate respiratory airway disease (Takeda and Akira, 2005). Lipopolysaccharide (LPS), a major component of the cell wall of Gram-negative bacteria, has been shown to induce ALI (Yu et al., 2014). Upon entry into the biological organism, LPS contributes to lung injury primarily through the perception of innate immune cells and initiates the secretion of inflammatory mediators. This process destroys the alveolar epithelial and endothelial barriers to impair the normal respiratory function of the host (Kolomaznik et al., 2017). Therefore, LPS-induced animal models of ALI are commonly used to explore the mechanisms of the lung inflammatory response.

Endoplasmic reticulum (ER) stress is defined as the accumulation of unfolded or misfolded proteins in the ER and the subsequent triggering of the unfolded protein response (UPR). It is facilitated by three transmembrane ER signaling proteins: pancreatic endoplasmic reticulum kinase (PERK), inositol-requiring enzyme 1 (IRE1), and activating transcription factor 6 (ATF6) (Hosoi and Ozawa, 2009). Accumulation of unfolded peptides diverts GRP78 away from these three “stress sensors” thus inducing changes in downstream signals such as XBP1, CHOP, and eIF2α, which leads to upregulation of various UPR-targeted genes to restore ER homeostasis (Wang et al., 2012). Recent studies have indicated that ER stress is involved in LPS-stimulated airway epithelial cells *in vitro* and activation of the ATF4/eIF2a/CHOP signaling axis to promote cell death (Kim et al., 2013). It is not coincidence that inhibition of NF-κB inflammatory signaling is effective in suppressing oxidative stress and ER stress to alleviate ALI (Yang et al., 2020). However, since the role of ER stress in LPS-induced lung inflammation has not been fully elucidated, there is still no suitable agent targeting ER stress for the treatment of ALI.

Traditional Chinese medicines are often remarkably effective in treating ALI/ARDS. Based on *in vivo* and *in vitro* results, a variety of natural metabolites with multiple anti-inflammatory activities and lung protective effects, such as flavonoids, alkaloids, and terpenoids, have been proposed for treating ALI (He et al., 2021). Studies have shown that *Bauhinia championii* (Benth.) Benth. (Leguminosea), extracts have antitumor, antioxidant, and ER stress-alleviating effects (Chen et al., 2020; Liao et al., 2016). Our previous study also demonstrated that extracts of *B. championii* alleviates neuronal apoptosis by modulating ER stress (Huang et al., 2022). A network analysis showed that flavonoids in *B. championii* have a potential anti-inflammatory function. In the present study, we extracted, isolated and characterized a class of polymethoxyflavones (PMFs) from *B. championii*. Our objectives were to explore the mitigating effects of PMFs on LPS-induced ALI and to explain the mechanism by which PMFs reduce apoptosis in lung cells by modulating endoplasmic reticulum (ER) stress in lung cells to reduce inflammatory responses and reactive oxygen species (ROS) accumulation.

## 2 Materials and methods

### 2.1 Drugs and reagents

The stem of *B. championii* was collected from Fujian Province (on 6 May 2021). It was identified and gifted by Professor Chen Jianzhong (Fujian University of Traditional Chinese Medicine). A voucher specimen was deposited in the Herbarium of Hangzhou Medical College.

Silica gel (100–200 mesh) was purchased from Qingdao Ocean Chemical Co., Ltd. (China). ODS-A-HG (50 μm) and the YMC-Pack ODS-A-HG column (250 mm × 10 mm i. d., 10 μm) were purchased from YMC. Co., Ltd. (Japan). Agilent ZORBAX Extend-C18 column (250 mm × 4.6 mm i. d., 5 μm) was purchased from Agilent Technologies, Inc. (Agilent, United States). Methanol, and formic acid (all LC-MS grade) were purchased from Thermo Fisher (United States). CD_3_Cl (D, 99.8% + 0.03 v/v TMS) was purchased from Tenglong Weibo Technology (China). The reference standard of sinensetin (No. FY1653-B) was purchased from Nantong Jingwei Biological Technology Co., Ltd. (China).

Malondialdehyde (MDA), glutathione peroxidase (GSH-Px), catalase (CAT) and hydroxyproline (HYP) detection kits were purchased from Nanjing Jiancheng Bioengineering Institute (China). The dihydroethidium (DHE) kit, TdT-mediated dUTP Nick-End Labeling (TUNEL) kit, BCA kit, ECL kit and Wright-Giemsa staining kit were obtained from Shanghai Biyuntian Biological Co., Ltd. (China). Primary antibodies targeting the following proteins were used: IKKα, IκB, p-IκB, NF-κB, p-NF-κB, PERK, p-PERK, eIF2α, p-eIF2α, JNK, and p-JNK (CST, United States), IRE1α, p-IRE1α, and GRP78 (Abcam, United States), ATF4, CHOP, TRAF2, and ATF6 (Proteintech Group, United States), and GAPDH (Hangzhou Xianzhi, China). HRP-conjugated goat anti-rabbit IgG (H + L) was purchased from Proteintech. Other reagents were purchased from Thermo Scientific (United States).

### 2.2 Purification of PMFs

Dried slices of *B. championii* were ground, and 1.5 kg of powder was extracted with 15 L MeOH under ultrasonication for 1 h at room temperature (repeated three times). The MeOH extract was concentrated under reduced pressure to yield a crude residue (248.8 g), which was suspended in water and successively partitioned with ethyl acetate and n-butyl alcohol. After concentration, the ethyl acetate fraction (93.6 g) was chromatographed over silica gel and eluted with gradient mixtures of petroleum ether/ethyl acetate (4:6–1:9) to yield 5 fractions. Fr. Four was rechromatographed by an ODS C18 column and eluted with gradient mixtures of MeOH/H_2_O (1:9–6:4) to afford 4.5 g PMFs. Appropriate quantities of PMFs was separated by prep-HPLC to afford compounds PMF1-5 (4, 12, 15, 7 and 5 mg, respectively). The purity of PMF1-5 was detected by the peak area normalization method using high-performance liquid chromatography (HPLC), all of which were greater than 98%.

### 2.3 Identification of the main metabolites in PMFs

Metabolites PMF 1–5 (2–5 mg) were dissolved in 0.6 mL CDCl_3_ and transferred to NMR tubes. 1H-NMR spectra (600 MHz) were recorded on a Bruker AV-600 spectrometer. Chemical shifts were recorded in δ units (ppm) and coupling constants (J) in Hz. HR-ESI-MS experiments were carried out using an Agilent 1260 HPLC/6,530 Q-TOF mass spectrometer. An Agilent ZORBAX Extend-C18 column (250 mm × 4.6 mm, i. d. 5 μm) was used for chromatographic separation. The mobile phase consisted of 0.1% formic acid in H_2_O (A) and methanol (B). The elution program was as follows: 0–5 min, 10% B; 5–30 min, 10%–20% B; 30–55 min, 20%–35% B; 55–70 min, 35%–55% B; 70–95 min, and 55%–65% B. The flow rate was 1 mL/min and the injection volume was 5 μL. The column compartment was maintained at 30°C. The mass spectrometry (MS) spectra were recorded under electrospray ionization (ESI) positive mode. The MS parameters were as follows: scan range, *m*/*z* 100–1,400; nitrogen flow rate, 8 L/min; drying gas temperature, 300°C; nebulizer pressure, 35 psi; capillary voltage, 3500 V. A collision energy of 30 eV was used in MS/MS.

### 2.4 HPLC analysis of PMFs

The HPLC analysis was performed on a Waters Acquity Arc ultra-high-performance liquid chromatography (UHPLC) system equipped with a Waters 2,998 photodiode array (PDA) detector. An Agilent ZORBAX Extend-C18 column (250 mm × 4.6 mm i. d. 5 μm) was used for the chromatographic separation. The mobile phase consisted of 0.1% formic acid in H_2_O (A) and methanol (B). The elution program was as follows: 0–2 min, 55% B; 2–17 min, 55%–70% B; 17–20 min, 70%–90% B; and 20–22 min, 90% B. The flow rate was 1 mL/min and the injection volume was 5 μL. The ultraviolet (UV) detection wavelengths were set at 332 nm for PMF 1, 320 nm for PMF 2, 327 nm for PMF 3, 334 nm for PMF 4 and 339 nm for PMF 5. The column compartment was maintained at 30°C.

The PMFs (2.04 mg) were dissolved in methanol in a 1 mL volumetric flask. Precisely 0.5 mL of the solution was transferred into a 25 mL volumetric flask and filled to the mark with methanol. The solution was filtered through a 0.22 μm polytetrafluoroethylene (PTFE) filter for the HPLC analysis.

The PMF 1–5 compounds were accurately weighed to be 1.62, 1.36, 1.57, 1.28, and 1.92 mg and dissolved in methanol in a 1 mL volumetric flask to make the standard stock solutions. Appropriate volumes of the stock solutions of PMF 1–5 were carefully transferred into a 10 mL volumetric flask to obtain a mixture of standard stock solutions of PMF 1–5 of 1.62, 27.2, 31.4, 12.8, and 1.92 μg/mL. Five calibration curves of PMF 1–5 were prepared withed six appropriate dilutions of the mix stock solution. An aliquot of 5 μL of the sample and standard solution was used for the HPLC analyses.

### 2.5 Predicting potential targets for MBC

The known metabolites in *B. championii* (MBC) were obtained by reading the literature and searching on the Chemistry Database (https://organchem.csdb.cn), and the known metabolites were entered into the Pubchem (https://pubchem.ncbi.nlm.nih.gov/) database to obtain their canonical simplified molecular-input line-entry system (SMILES). These were then sequentially imported into SwissADEM (http://www.swissadme.ch/) and screened for active metabolites with scores for gastrointestinal (GI) absorption of “high” and drug likeness with at least two “Yes”. Subsequently, the canonical SMILES of the screened metabolites were entered into the SwissTargetPrediction (http://www.swisstargetprediction.ch/) to predict the targets, and a “probability>0” was used as the filtering condition to obtain the potential targets of the Nine Dragons Vine.

### 2.6 Predicting potential targets for ALI

The OMIM (https://www.omim.org/), GeneCards (https://www.genecards.org/) and DisGeNET (https://www.disgenet.org/) databases were searched using the keyword “acute lung injury”. To ensure the reliability of the targets, the targets obtained from the three databases were exported after removing the duplicate genes to create a database of targets related to acute lung injury.

### 2.7 Identifying core targets and the protein-protein interaction (PPI) network construction

The targets of acute lung injury and the targets of MBC were compared using an online tool (http://www.liuxiaoyuyuan.cn/) to obtain the intersection targets of MBC and acute lung injury. We set the species as “*Homo sapiens*” and uploaded the intersecting targets into the STRING (https://string-db.org/) database for the PPI network construction. Cytoscape 3.9.1 was utilized for mapping.

### 2.8 GO and KEGG enrichment analysis

“*H. sapiens*” was set as the species, a Geno Ontology (GO) and Kyoto Encyclopedia of Genes and Genomes (KEGG) enrichment analysis were performed on the intersecting targets using the Database for Annotation, Visualization, and Integrated Discovery (DAVID) (https://david.ncifcrf.gov/), and the top 20 enrichment results were plotted as visualized bubble plots.

### 2.9 Molecular docking

The relevant targets of the endoplasmic reticulum stress pathway were obtained using the GeneCards (https://www.genecards.org/) database. The intersection was found using the targets of five PMFs, the targets of the endoplasmic reticulum stress pathway, and the targets of acute lung injury to obtain the key targets of PMFs for the treatment of acute lung injury through endoplasmic reticulum stress. The key targets were entered into the Protein Data Bank (PDB) (https://www.rcsb.org/) database to obtain their structures and molecularly docked with PMFs using Autodock to observe the binding energies of each group.

### 2.10 Animals and treatments

Seventy-two specific pathogen-free (SPF) male C57BL/6 mice, weighing 16–20 g, were purchased from the Zhejiang Provincial Laboratory Animal Center. The mice were housed in a temperature-controlled environment (22°C ± 2°C) under humidity-controlled conditions (55% ± 5%) with a 12 h light/dark cycle and free access to water and food. Mice were housed in cages (six mice per cage) and all mice were acclimatized to the living environment for 3°days before the experiment. All animal experiments in this study were approved by the Institutional Animal Care and Use Committee. Institutional Animal Care guidelines issued by the Chinese Ministry of Science and Technology were followed. All animal experiments were performed according to the protocol approved by the Experimental Animal Ethics Committee (2021-162) of Hangzhou Medical College.

Mice were randomly divided into six groups (12 mice per group): (i) mice in the control group received 50 μL of 0.9% saline intranasally to mimic nasal administration, and gavaged with 0.5% CMC-Na to simulate oral administration; (ii) mice in the LPS group were given 20 μg of LPS dissolved in 50 μL saline intranasally on the fifth day, and simulated gavaged with 0.5% CMC-Na on day 1, day 3, and 4 h after LPS treatment; (iii) mice in the LPS + Dex group received LPS and 5 mg/kg of dexamethasone was given intraperitoneally 4 h after LPS administration as well as 1 day before LPS treatment; (iv) mice in the low-dose PMF group [LPS + PMFs(L)] were given PMFs at a dose of 100 mg/kg by gavage on day 1, day 3, and 4 h after LPS treatment; (v) mice in the medium-dose PMF group [LPS + PMFs(M)] were given PMFs at a dose of 300 mg/kg by gavage on day 1, day 3, and 4 h after LPS treatment; and (vi) mice in the high-dose PMF group [LPS + PMFs(H)] were given PMFs at a dose of 900 mg/kg by gavage on day 1, day 3, and 4 h after LPS treatment. The gavage volume for all animals was 0.2 mL per 10 g body weight. At 24 h after LPS administration, all mice were weighed, anesthetized, and executed. Lung wet and dry weight were recorded from six animals in each group; the lungs from three animals in each group were used for histological examination with alveolar lavage; the lung tissues of the remaining three mice from each group were used for Western blot and PCR assays. The detailed experimental schedule is shown in Figure 3A.

### 2.11 Collection of BALF and production of lung tissue slices

At 24 h after LPS treatment, mice in each group were executed by cervical dislocation. Then the thoracic cavity of the sacrificed mice was clipped, and when the trachea was exposed, a T-shaped incision was cut in the trachea, and the line trachea was inserted. In addition, the left lung was ligated at the left main bronchus. Finally, 1.5 mL of physiological saline was used to irrigate the right lung three times. The recovered fluid (first >80%) was used as bronchoalveolar lavage fluid (BALF).

The left lung was cut and collected after lavage and fixed in 10% formalin overnight. The next day, the fixed lungs were rinsed with water and dehydrated with serially diluted alcohol. Paraffin-embedded samples were sectioned at a thickness of 4–5 μm using sledge microtome.

### 2.12 BALF analysis

BALF (50 μL) was observed under a microscope and 500 μL BALF was used for antioxidant index detection. The remaining lavage fluid was centrifuged at 1,200 rpm for 5 min, and the supernatant was discarded. The cell precipitates were stained with Wright-Giemsa (Item No. C0131) staining solution and applied to the cell counting plates. Total cells, macrophages and neutrophils were counted under an optical microscope.

### 2.13 Antioxidant indices

The collected BALF was centrifuged at 1,200 rpm. The supernatants were used for the GSH-Px, CAT, MDA, and HYP assays after quantification of the total protein by a BCA kit (Item No. P0009). Samples treated with a GSH-Px kit (Item No. A005-1), CAT kit (Item No. A007-1), MDA kit (Item No. A003-1), and HYP kit (Item No. A030-2) were analyzed using a microplate reader at 412, 240, 532, and 550 nm, respectively. The levels of GSH-Px, CAT, MDA, and HYP in each sample were also calculated according to the colorimetric method following the manufacturer’s instructions.

### 2.14 H&E staining and immunohistochemistry

Lung slices were subjected to xylene deparaffinization and hydration with graded concentrations of ethanol. Slices were stained with hematoxylin, differentiated with 1% ethanol hydrochloride, and stained with 1% weak aqueous ammonia and eosin. Histopathology was observed with a microscope (Leica DM4000, Germany) by a pathologist. The degree of lung injury was demonstrated by the alveolar score as previous described (Wei et al., 2022). For immunohistochemistry, slices were also subjected to immunohistochemistry analysis. Endogenous peroxidase activity was quenched with 3% hydrogen peroxide. After washing with PBS, sections were blocked with 1% bovine serum albumin for 1 h at room temperature and then incubated with anti-GRP78 overnight at 4°C. The next day, sections were washed with PBS, followed by incubation with HRP-conjugated secondary antibody for 1 h. Next, tissue sections were incubated with diaminobenzidine, counterstained with hematoxylin, dehydrated, and mounted at room temperature. Sections were observed and imaged using an optical microscope. Integrated optical density (IOD) values were calculated using ImageJ (version 1.51).

### 2.15 TUNEL staining

A TUNEL kit (Item No. C1089) was used to detect apoptosis in lung sections following the manufacturer’s instructions. Briefly, paraffin sections were defatted and gradually rehydrated in xylene and a series of concentrations of ethanol. Then slices were incubated with proteinase K at 37°C for 30 min, followed by incubation with TUNEL reaction solution at 37°C for 1 h in the dark. Finally, samples were incubated with DAPI (Invitrogen, United States) for 5 min at room temperature to visualize the nuclei. Images were acquired using a fluorescence microscope (Olympus X51, Japan). TUNEL-positive cells were counted using ImageJ.

### 2.16 DHE staining

DHE staining was performed to measure ROS generation in lung tissues. Paraffin sections with a thickness of 5 μm were incubated with DHE solution (Item No. S0063) for 30 min at 37°C in the dark. Images were acquired using a fluorescence microscope and the fluorescence intensity of positive cells (red staining) was quantified using ImageJ.

### 2.17 Quantitative real-time PCR (RT-qPCR)

Lung tissues were cut into pieces, and total RNA was extracted using TRIzol^®^ reagent. Prime Script RT Master Mix (Takara, Japan) was used to reverse transcribe RNA and TB Green^®^ Premix Ex Taq™ (Takara, Japan) was used for the quantitative real-time PCR. Fusion curve analysis confirmed the specificity of PCR products. Relative quantification of gene expression was performed using the 2^−ΔΔCt^ method. Gene expression was normalized to β-actin. All primers (IL-6: forward, 5′-TCC​TAC​CCC​AAT​TTC​CAA​TGC​T-3′, reverse, 3′-TGG​TCT​TGG​TCC​TTA​GCC​AC-5′; IL-1β: forward, 5′-TGC​CAC​CTT​TTG​ACA​GTG​ATG-3′, reverse, 3′-ATG​TGC​TGC​TGC​GAG​ATT​TG-5′; TNF-α: forward, 5′-CCA​CGT​CGT​AGC​AAA​CCA​CC-3′, reverse, 3′-CCC​TTG​AAG​AGA​ACC​TGG​GAG-5′; IFN-γ: forward, 5′-GAG​GTC​AAC​AAC​CCA​CAG​GT-3′, reverse, 3′-GGG​ACA​ATC​TCT​TCC​CCA​CC-5′; β-actin: forward, 5′-AGG​GAA​ATC​GTG​CGT​GAC​AT-3′, reverse, 3′-GGA​AAA​GAG​CCT​CAG​GGC​AT-5′) were designed using GenBank (https://www.ncbi.nlm.nih.gov/genbank/).

### 2.18 Western blot analysis

Lung tissue was homogenized in RIPA buffer and then centrifuged at 12,000 × g for 15 min at 4°C. The pellet was discarded and the total protein concentration in the supernatant was determined using a BCA kit. Proteins were separated by SDS-PAGE and transferred to polyvinylidene difluoride membranes, which were blocked in 5% skimmed milk for 30 min and incubated with primary antibodies at 4°C overnight. The immunoreactive protein bands were detected using a Bio-Rad ChemiDoc MP system (Bio-Rad, United States) after dropwise addition of ECL developer.

### 2.19 Statistical analysis

Statistical analysis was performed using GraphPad Prism 8. Values are expressed as mean ± standard deviation (SD), and *P* values less than 0.05 were considered significant. Two-tailed Student’s t-test and one-way analysis of variance (ANOVA) followed by Dunnett’s *post hoc* test were used to compare between two groups and among three or more groups, respectively.

## 3 Results

### 3.1 Network analysis of MBC for treating ALI

**FIGURE 1:**
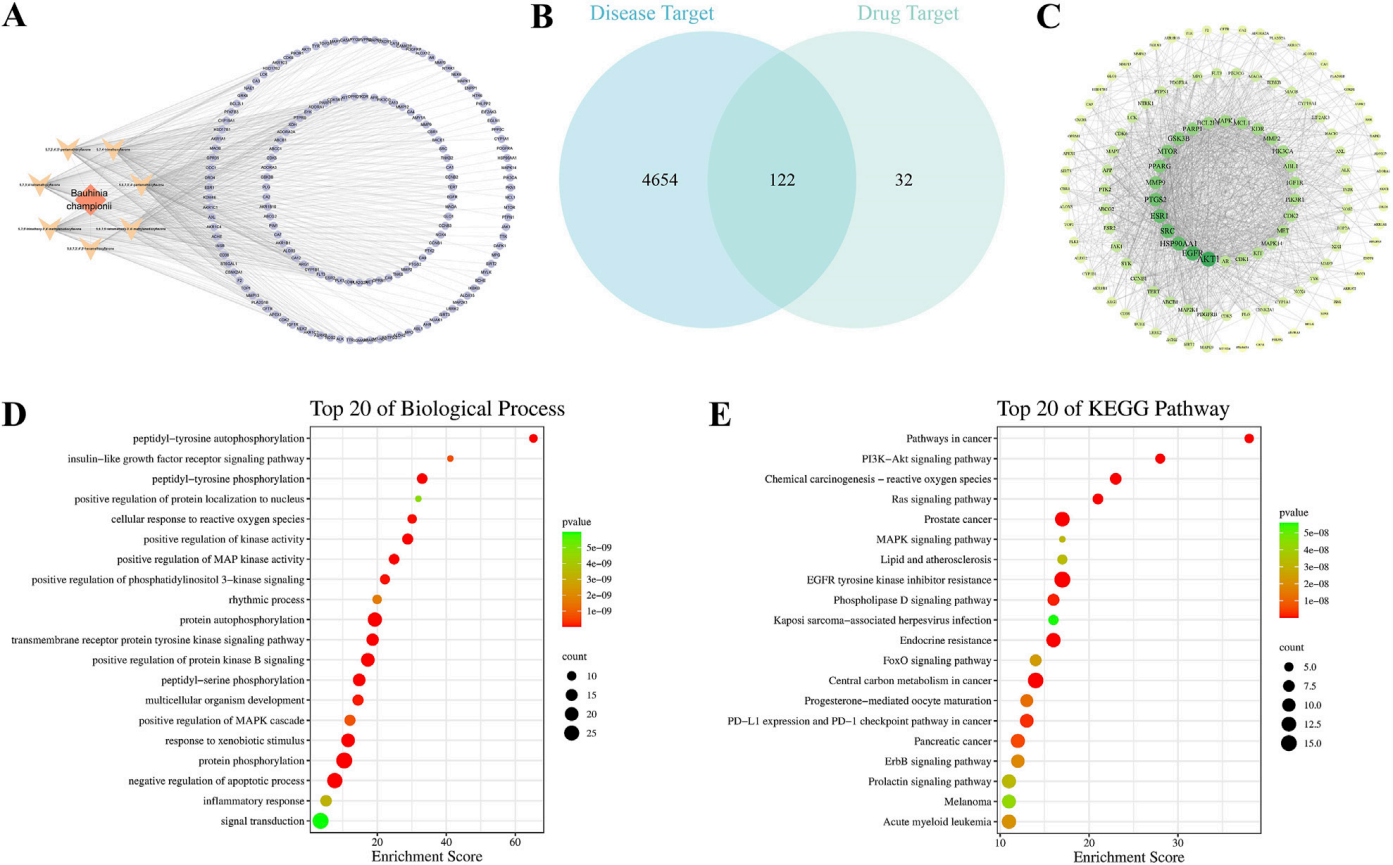
Network pharmacological analysis of *Bauhinia championii* for acute lung injury. **(A)** *Bauhinia championii*-Active Ingredient-Target Network, diamond represents the botanical drug, V-shape represents the active ingredient, and circle represents the target. **(B)** The Venn diagram of *Bauhinia championii’s* target and the target of acute lung injury, and the overlapped part represents the potential target of *Bauhinia championii* for the treatment of acute lung injury. **(C)** Protein Interaction Network of the 122 overlapped targets, and the nodes’ sizes and colors reflect the Degree size, larger nodes and darker colors represent higher Degree values. **(D)** GO (BPs) enrichment analysis of 122 overlapping targets. **(E)** KEGG pathway enrichment analysis of 122 overlapping targets.

To predict the active ingredients in *B. championii* and their molecular targets in the treatment of ALI, we conducted a network analysis using *in silico* simulations. Based on literature and Chemistry Database search, a total of 17 metabolites were identified in *B. championii*, among which 7 flavonoids and 1 organic acid were screened by SwissADEM as potential active metabolites (Table 1). The 7 flavonoids, including 5, 6, 7, 3′,4′-pentamethoxyflavone, 5, 6, 7, 3′, 4′, 5′-hexamethoxyflavone, 5, 7, 3′, 4′, 5′-pentamethoxyflavone, 5, 6, 7, 5′-tetramethoxy-3′, 4′-methylenedioxyflavone, 5, 7, 5′-trimethoxy-3′, 4′-methylenedioxyflavone, 5, 7, 4′-trimethoxyflavone and 5, 7, 3′, 4′-telramethoxyflavone, considered as active metabolites predicted a total of 154 targets (Figure 1A). A total of 4,776 targets related to ALI were screened, of which 122 targets were also present in the MBC’s targets (Figure 1B), and these 122 targets were considered as potential targets of MBC for the treatment of ALI. The STRING platform was utilized to construct the PPI network of potential targets, and based on the screening of network topological properties, the top 20 nodes in the network in terms of degree were found to include AKT1, BCL2L1, and PIK3R1 (Figure 1C). In addition, the results of the GO and KEGG enrichments showed that MBC could treat ALI through anti-inflammatory, antioxidant and anti-apoptotic approaches (Figures 1D,E).

**TABLE 1:** The chemical names, Molecular formulas and SMILES of 8 potential active metabolites found in *Bauhinia championii*.

Chemical name	Molecular formula	SMlLES	References
5, 6, 7, 3′, 4′-pentamethoxyflavone	C_20_H_20_O_7_	COC1 = C(C=C(C=C1)C2 = CC(=O)C3 = C(C(=C(C=C3O2)OC)OC)OC)OC	Cai-Xia et al. (2016), Chen et al. (1984), Faqueti et al. (2017), Zhou et al. (2009)
5, 6, 7, 3′, 4′, 5′-hexamethoxyflavone	C_21_H_22_O_8_	COC1 = CC(=CC(=C1OC)OC)C2 = CC(=O)C3 = C(C(=C(C=C3O2)OC)OC)OC	Bai et al. (2005), Cai-Xia et al. (2016), Chen et al. (1984), Faqueti et al. (2017)
5, 7, 3′, 4′, 5′-pentamethoxyflavone	C_20_H_20_O_7_	COC1 = CC2 = C(C(=C1)OC)C (=O)C=C(O2)C3 = CC(=C(C(=C3)OC)OC)OC	Cai-Xia et al. (2016), Chen et al. (1984)
5, 6, 7, 5′-tetramethoxy-3′, 4′-methylenedioxyflavone	C_20_H_18_O_8_	COC1 = CC(=CC2 = C1OCO2)C3 = CC(=O)C4 = C(C(=C(C=C4O3)OC)OC)OC	Bai et al. (2005), Cai-Xia et al. (2016), Chen et al. (1984)
5, 7, 5′-trimethoxy-3′, 4′-methylenedioxyflavone	C_19_H_16_O_7_	COC1 = CC2 = C(C(=C1)OC)C (=O)C=C(O2)C3 = CC4 = C(C(=C3)OC)OCO4	Chen et al. (1984)
5, 7, 4′-trimethoxyflavone	C_18_H_16_O_5_	COC1 = CC = C(C=C1)C2 = CC(=O)C3 = C(O2)C=C(C=C3OC)OC	Cai-Xia et al. (2016)
5, 7, 3′, 4′-tetramethoxyflavone	C_19_H_18_O_6_	COC1 = C(C=C(C=C1)C2 = CC(=O)C3 = C(O2)C=C(C=C3OC)OC)OC	Cai-Xia et al. (2016), Chen et al. (1984)
gallic acid	C_7_H_6_O_5_	C1 = C(C=C(C(=C1O)O)O)C (=O)O	Bai et al. (2005)

### 3.2 Purification and identification of PMFs

**FIGURE 2:**
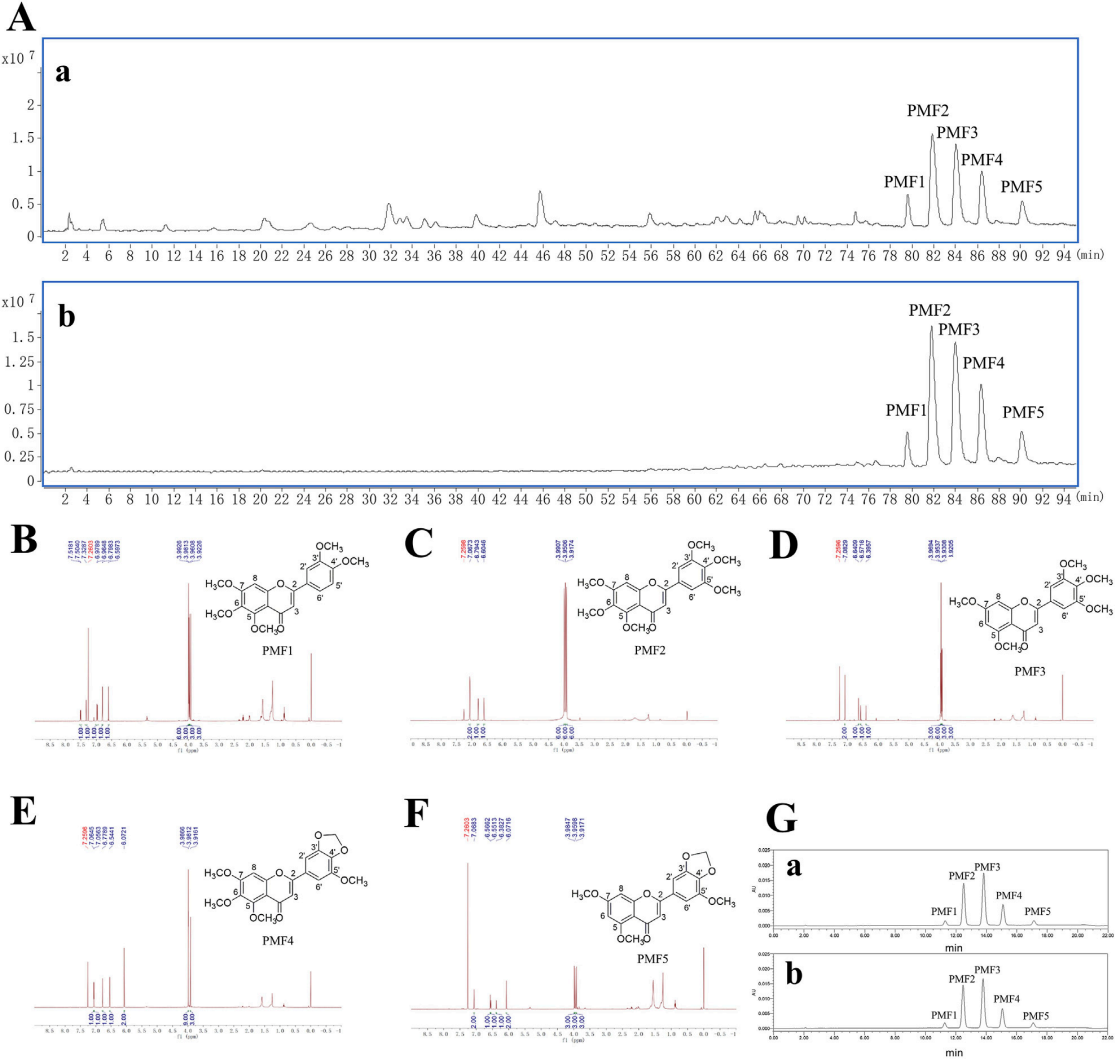
Identification and quantitative analysis of PMFs. **(A)** Total ion chromatograms of the ethyl acetate fraction (a) and PMFs (b) from *Bauhinia championii* in positive mode. **(B–F)** ^1^H-NMR spectras and chemical structures of PMF 1–5 separated from PMFs (PMFs 1, 5, 6, 7, 3′, 4′-pentamethoxyflavone. PMFs 2, 5, 6, 7, 3′, 4′, 5′-hexamethoxyflavone. PMFs 3, 5, 7, 3′, 4′, 5′-pentamethoxyflavone. PMFs 4, 5, 6, 7, 5′-tetramethoxy-3′, 4′-methylenedioxyflavone. PMFs 5, 5, 7, 5′-trimethoxy-3′, 4′-methylenedioxyflavone). **(G)** HPLC-DAD chromatograms of mixed standards of PMF 1–5 (a) and PMFs sample (b).

To elucidate the compositional profile of PMFs extracted from Bauhinia championii in this study, ^1^H-NMR spectras and LC-MS techniques were employed to perform qualitative and quantitative analyses of these compounds. PMFs were separated from Bauhinia championii. The HPLC-DAD-Q-TOF-MS analysis of PMFs (Supplementary Figure S1) and the ethyl acetate fraction of *B. championii* were shown in Figure 2A.

The structure of PMF 1 was determined to be 5, 6, 7, 3′, 4′-pentamethoxyflavone (Figure 2B). It showed a molecular formula of C_20_H_20_O_7_ from its positive HR-ESI-MS data (*m*/*z* 372.1215; calc. for C_20_H_20_O_7_, 372.1209, Diff −0.6 mDa); MS/MS: *m*/*z* 373([M + H]^+^), 357([M + H-CH_4_]^+^), 343([M + H-2CH_3_^•^]^+^), 329([M + H-CO_2_]^+^), 312([M + H-CH_3_^•^-CO-H_2_O]^+^), 297([M + H-2CH_3_^•^-CO-H_2_O]^+^) (Supplementary Figures S2, S3); ^1^H NMR (CDCl_3_) δ: 3.92 (3H, s, 3′-OMe), 3.96 (3H, s, 4′-OMe), 3.98 (3H, s, 5-OMe), 3.99 (6H, s, 6-OMe, 7-OMe), 6.59 (1H, s, H-3), 6.79 (1H, s, H-8), 6.97 (1H, d, J = 8.46 Hz, H-5′), 7.32 (1H, s, H-2′), 7.51 (1H, dd, J = 8.46 Hz, H-6′). The above data were consistent with the reference standard of sinensetin and the literature data (Cai-Xia et al., 2016; Chen et al., 1984; Faqueti et al., 2017; Zhou et al., 2009).

The structure of PMF 2 was determined to be 5, 6, 7, 3′, 4′, 5′-hexamethoxyflavone (Figure 2C). It showed a molecular formula of C_21_H_22_O_8_ from its positive HR-ESI-MS data (*m*/*z* 402.1317; calc. for C_21_H_22_O_8_, 402.1315, Diff −0.27 mDa); MS/MS: *m*/*z* m/z 403 [M + H]^+^, 387([M + H-CH_4_]^+^), 373([M + H-2CH_3_^•^]^+^), 359([M + H-CO_2_]^+^), 345([M + H-2CH_3_^•^-CO]^+^), 327([M + H-2CH_3_^•^-CO-H_2_O]^+^), 312([M + H-3CH_3_^•^-CO-H_2_O]^+^), 298([M + H-CH_3_^•^-CO-H_2_O-CO_2_]^+^) (Supplementary Figures S4, S5); ^1^H NMR (CDCl_3_) δ: 3.92 (6H, s, 3′-OMe, 5′-OMe), 3.95 (6H, s, 6-OMe, 4′-OMe), 3.99 (6H, s, 5-OMe, 7-OMe), 6.60 (1H, s, H-3), 6.79 (1H, s, H-8), 7.06 (2H, s, H-2′, H-6′). The above data were consistent with the literature data (Bai et al., 2005; Cai-Xia et al., 2016; Chen et al., 1984; Faqueti et al., 2017).

The structure of PMF 3 was determined to be 5, 7, 3′, 4′, 5′-pentamethoxyflavone (Figure 2D). It showed a molecular formula of C_20_H_20_O_7_ from its positive HR-ESI-MS data (*m*/*z* 372.1210; calc. for C_20_H_20_O_7_, 372.1209, Diff −0.09 mDa); MS/MS: *m*/*z* 373 [M + H]^+^, 357([M + H-CH_4_]^+^), 343([M + H-2CH_3_^•^]^+^), 329([M + H-CO_2_]^+^), 312([M + H-CH_3_^•^-CO-H_2_O]^+^) (Supplementary Figures S6, S7); ^1^H NMR (CDCl_3_) δ: 3.92 (3H, s, 3′-OMe), 3.93 (3H, s, 5′-OMe), 3.95 (6H, s, 5-OMe, 7-OMe), 3.97 (3H, s, 4′-OMe), 6.39 (1H, s, H-6), 6.57 (1H, s, H-8), 6.64 (1H, s, H-3), 7.08 (2H, s, H-2′, H-6′). These data were consistent with the literature data (Cai-Xia et al., 2016; Chen et al., 1984).

The structure of PMF 4 was determined to be 5, 6, 7, 5′-tetramethoxy-3′, 4′-methylenedioxyflavone (Figure 2E). It showed a molecular formula of C_20_H_18_O_8_ from its positive HR-ESI-MS data (*m*/*z* 386.1005; calc. for C_20_H_18_O_8_, 386.1002, Diff −0.3 mDa); MS/MS: *m*/*z* 387([M + H]^+^), 371([M + H-CH_4_]^+^), 357([M + H-2CH_3_^•^]^+^), 343([M + H-CO_2_]^+^), 329([M + H-2CH_3_^•^-CO]^+^) (Supplementary Figures S8, S9); ^1^H NMR (CDCl_3_) δ: 3.91 (3H, s, 5′-OMe), 3.98 (9H, s, 5-OMe, 6-OMe, 7-OMe), 6.07 (2H, s, OCH_2_O), 6.54 (1H, s, H-3), 6.77 (1H, s, H-8), 7.05 (^1^H, s, H-2′), 7.06 (1H, s, H-6′). These data were consistent with the literature data (Bai et al., 2005; Cai-Xia et al., 2016; Chen et al., 1984).

The structure of PMF 5 was determined to be 5, 7, 5′-trimethoxy-3′, 4′-methylenedioxyflavone (Figure 2F). It showed a molecular formula of C_19_H_16_O_7_ from its positive HR-ESI-MS data (*m*/*z* 356.0898; calc. For C_19_H_16_O_7_, 356.0896, Diff −0.17 mDa); MS/MS: *m*/*z* 357([M + H]^+^), 342([M + H-CH_3_^•^]^+^), 313([M + H-CO_2_]^+^), 285([M + H-2CH_3_^•^-CO-CH_2_]^+^) (Supplementary Figures S10, S11); ^1^H NMR (CDCl_3_) δ: 3.91 (3H, s, 5′-OMe), 3.95 (3H, s, 5-OMe), 3.98 (3H, s, 7-OMe), 6.07 (2H, s, OCH_2_O), 6.38 (1H, s, H-6), 6.55 (1H, s, H-8), 6.56 (1H, s, H-3), 7.06 (2H, s, H-2′, H-6′). The above data were consistent with the literature data (Chen et al., 1984).

Five calibration curves were constructed by plotting the peak area of analytes against concentration (μg/mL) for PMF1-5. The regression equations, correlation coefficients, linearity ranges, and PMF1-5 contents in PMFs are shown in Table 2. The calibration curves exhibit good linearity with correlation coefficients higher than 0.9999. The contents of PMF1-5 were between 2.13 and 36.59 g/100 g. The HPLC chromatogram of PMFs and the standards are shown in Figure 2G.

**TABLE 2:** Linearity and contents of PMF1-5 in PMFs.

Compound	Regressive equation	R^2^	Linear range (μg/mL)	Formula	t_R_ (min)	Content (g/100g)
PMF1	y = 22603x−80.53	0.9999	0.05–1.62	C_20_H_20_O_7_	11.259	2.13
PMF2	y = 13226x+1718.3	1	0.85–27.2	C_21_H_22_O_8_	12.470	34.31
PMF3	y = 14034x+2475.2	1	0.98–31.4	C_20_H_20_O_7_	13.774	36.59
PMF4	y = 13814x+675.98	1	0.40–12.8	C_20_H_18_O_8_	15.038	14.66
PMF5	y = 22551x−107.59	1	0.06–1.92	C_19_H_16_O_7_	17.044	2.49

### 3.3 Effects of PMFs in LPS-induced acute lung injury

**FIGURE 3:**
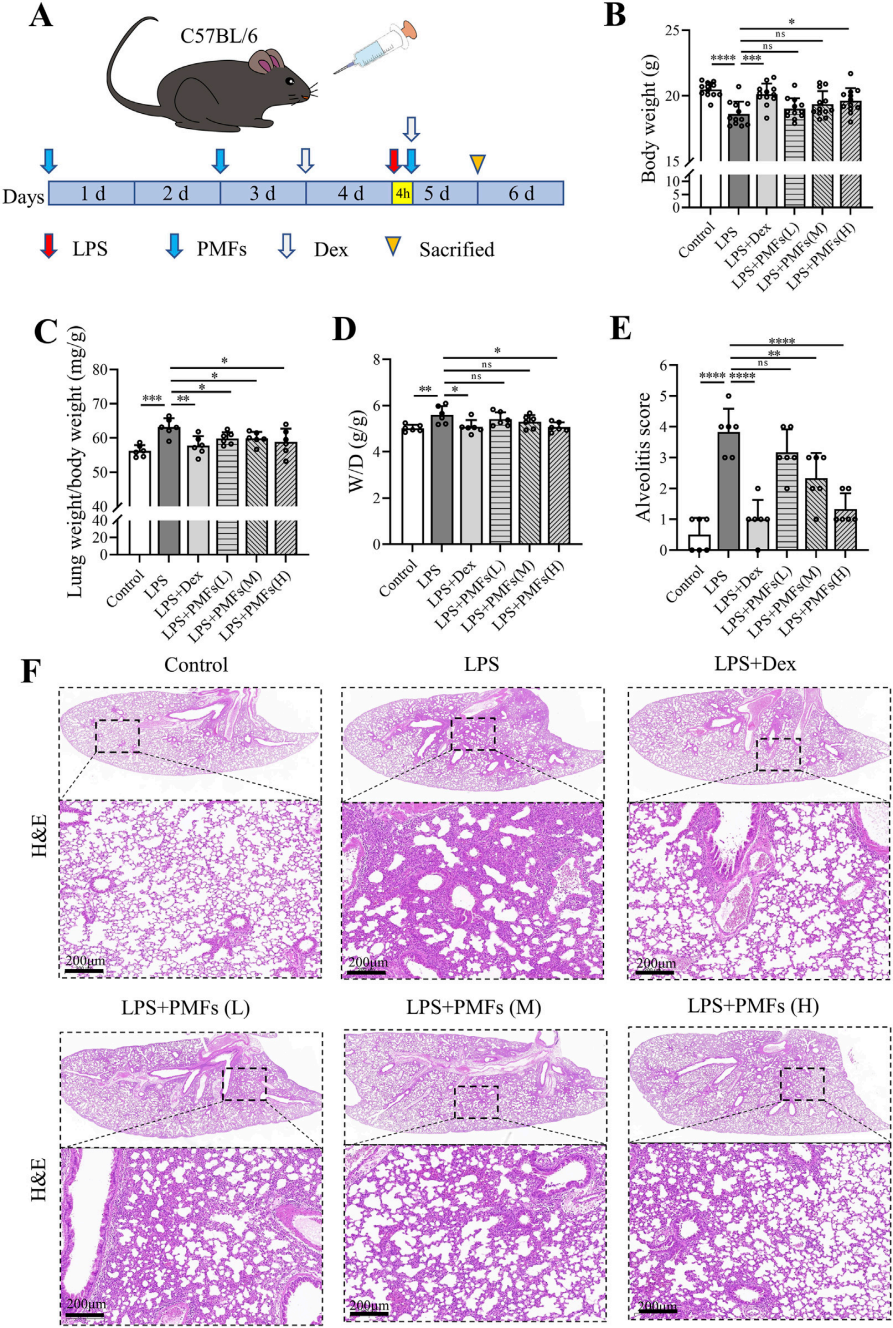
Effects of PMFs in LPS-induced acute lung injury. **(A)** Experimental schedule for study design. **(B)** Body weight (n = 12). **(C)** Lung weight/body weight ratio (n = 6). **(D)** Lung wet-dry weight ratio (W/D) (n = 6). Scale bar = 200 μm. **(E)** Alveolitis score for evaluation of lung lesion. **(F)** H&E staining to detect histological changes in the lung tissues of mice. Data are presented as mean ± SD, *p < 0.05, **p < 0.01, ***p < 0.001, ****p < 0.0001.

To investigate the effects of PMFs on LPS-induced ALI, we used three different doses of PMFs to treat LPS-induced ALI model mice. Dex was used as a positive control. At 24 h after the intranasal administration of 20 μg of LPS, the mice in the LPS group showed significant loss of body weight. However, PMFs counteracted LPS-induced toxicity, especially in the LPS + PMFs(H) group (Figure 3B). Based on the lung wet/dry weight ratio (W/D) and the lung weight/body weight ratio, it was hypothesized that LPS increased the lung weight of mice and caused pulmonary edema (Li et al., 2021), whereas PMFs effectively decreased the lung weight/body weight ratio. PMFs at a dose of 900 mg/kg significantly attenuated LPS-induced pulmonary edema, which was similar to the effect of Dex treatment (Figures 3C,D). Histological examination showed that LPS induced severe granulocyte infiltration in the lung tissue and destroyed the alveolar structure, causing extensive lung tissue damage. PMFs reduced LPS-induced inflammatory cell infiltration and lung tissue damage in a dose-dependent manner (Figures 3E,F).

### 3.4 Effects of PMFs on LPS induced-lung inflammation

**FIGURE 4:**
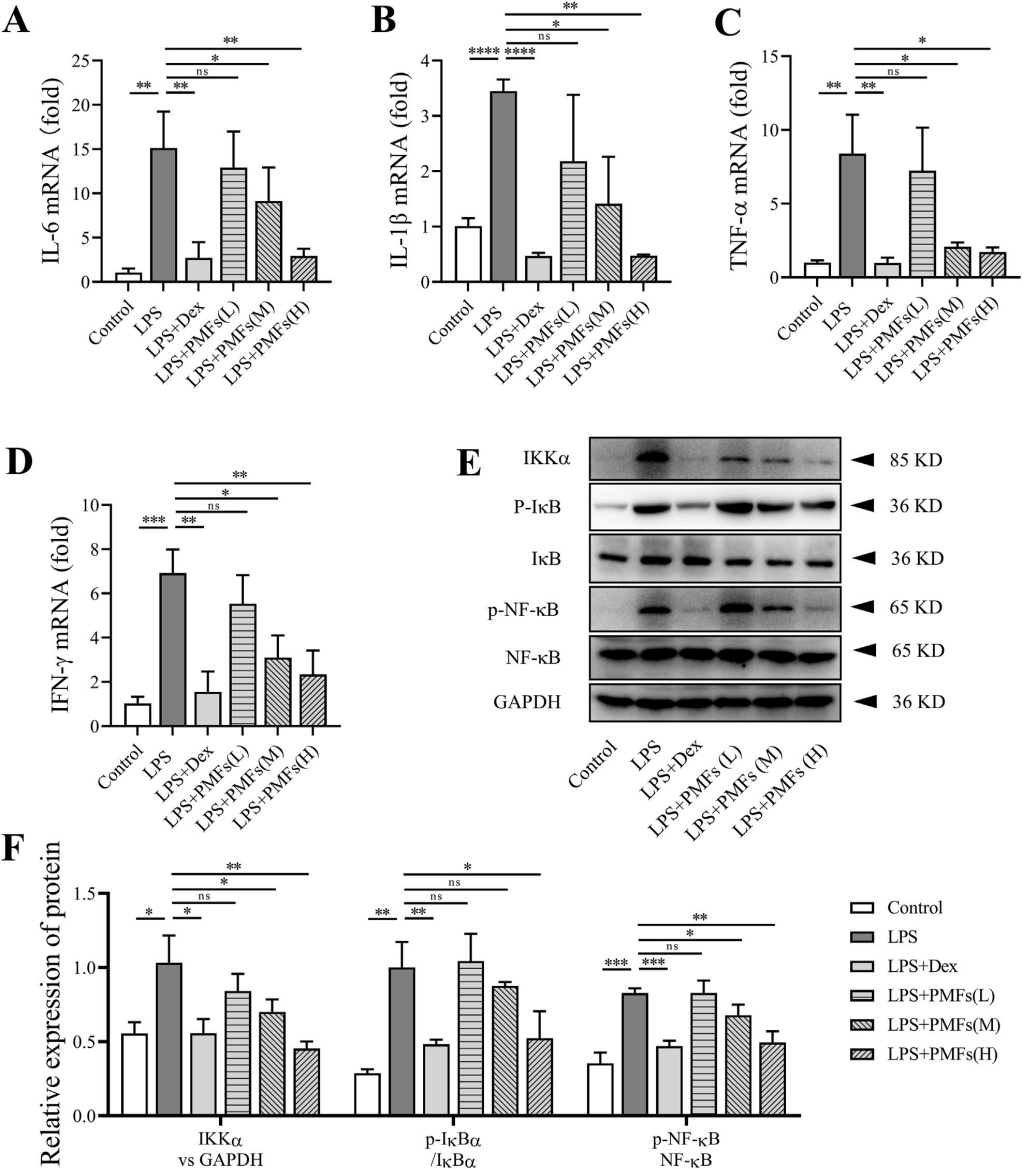
The effects of PMFs in LPS induced lung inflammation. Data are presented as mean ± SD; n = 3, *p < 0.05, **p < 0.01, ***p < 0.001, ****p < 0.0001. **(A–D)** IL-6, IL-1β, TNF-α and IFN-γ transcript in C57BL/6 mice and normalized to β-Actin expression. **(E)** Western blot analysis for Ikkα, IκB, p- IκB, NF-κB, p-NF-κB and GAPDH. **(F)** Quantitative analysis for p-IκB/IκB, p-NF-κB/NF-κB and Ikkα expression.

To elucidate the mechanism by which PMFs alleviate the LPS-induced inflammatory response in the lungs, we determined the expression levels of pro-inflammatory factors in lung tissues. The results showed that LPS raised the mRNA levels of IL-6, IL-1β, TNF-α, and IFN-γ. The mRNA levels of pro-inflammatory factors in lung tissues were significantly reduced when mice were treated with PMFs at doses of 300 and 900 mg/kg (Figures 4A–D). Western blot analysis showed that LPS induced the release of pro-inflammatory factors through activation of the NF-κB signaling pathway. PMFs at doses of 300 and 900 mg/kg decreased the expression of IKKα, reduced the phosphorylation of IκB, and blocked the activation of NF-κB signaling to inhibit the inflammatory signaling pathway in the lung tissues of mice (Figures 4E,F). PMFs at a dose of 900 mg/kg could achieve a similar effect as Dex in reducing the inflammatory response in the lungs of mice.

### 3.5 Modulation of oxidative stress in the lung by PMFs

**FIGURE 5:**
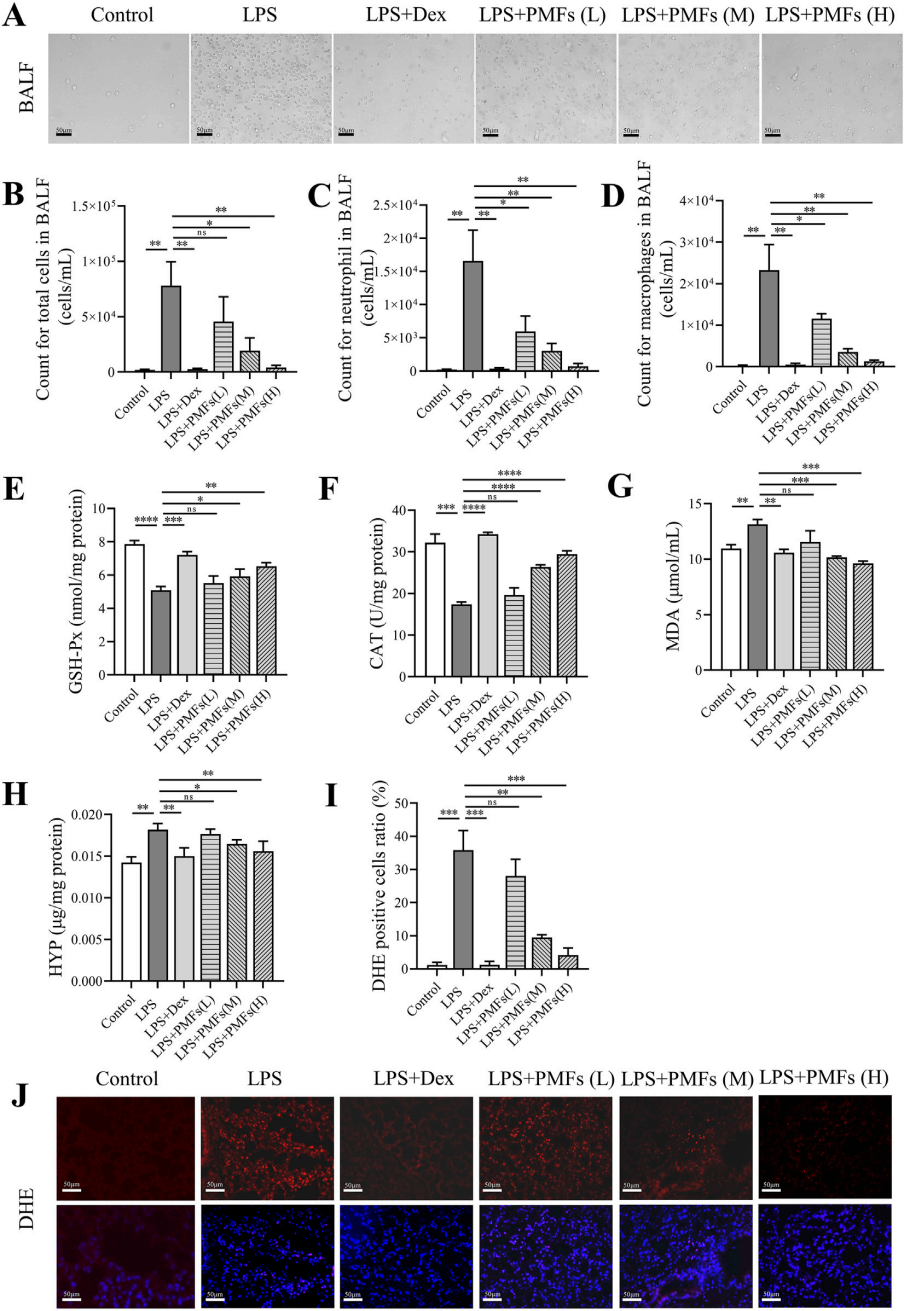
The effects of PMFs on regulating oxidative stress. Data are presented as mean ± SD; n = 3, *p < 0.05, **p < 0.01, ***p < 0.001, ****p < 0.0001. **(A)** The image of BALF. Scale bar = 20 μm. **(B)** The count for cells in BALF. **(C)** The count for neutrophil in BALF. **(D)** The count for macrophages in BALF. **(E)** The level of GSH-Px in BALF. **(F)** The level of CAT in BALF. **(G)** The level of MDA in BALF. **(H)** The level of HYP in BALF. **(I)** Quantitative analysis for DHE positive cell ratio. **(J)** Representative DHE staining of lung. Scale bar = 50 μm.

On examining BALF, we found that LPS increased the permeability of lung tissues, enabling the presence of many cells in the BALF. By sorting and counting the cells in BALF, we found that LPS induced aggregation of neutrophils and macrophages in BALF, whereas Dex greatly reduced the aggregation of inflammatory cells in BALF. Similarly, PMFs were able to reduce the LPS-induced increase in inflammatory cells in BALF (Figures 5A–D). LPS-induced aggregation of inflammatory cells within BALF was also able to promote oxidative stress. As shown in Figures 5E–H, LPS decreased GSH-Px and CAT levels and increased MDA and HYP levels in BALF. In contrast, after treatment with PMFs at doses of 300 and 900 mg/kg, GSH-Px and CAT levels were significantly restored, while the levels of MDA and HYP in BALF were significantly diminished. DHE staining of lung tissues showed that PMFs at doses of 300 and 900 mg/kg markedly inhibited the LPS-induced increase in ROS levels in lung tissues (Figures 5I,J).

### 3.6 Molecular docking for PMFs acting on ER stress targets

**FIGURE 6:**
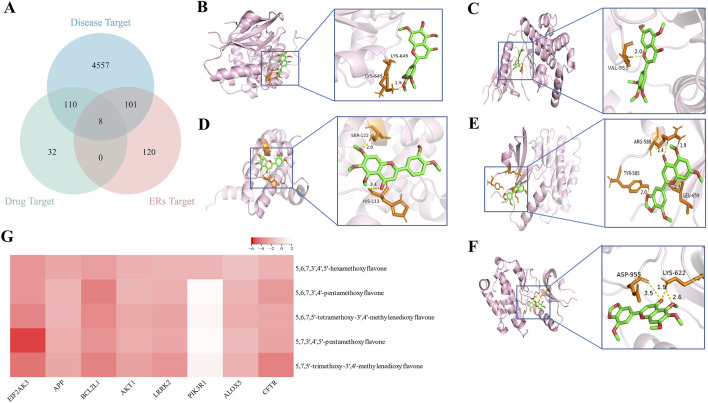
Molecular docking. **(A)** The Venn diagram of the targets of five PMFs, the targets of acute lung injury, and targets of the endoplasmic reticulum stress pathway. **(B)** 5, 6, 7, 3′, 4′, 5′-hexamethoxyflavone binds EIF2AK3. **(C)** 5, 7, 3′, 4′, 5′-pentamethoxyflavone binds EIF2AK3. **(D)** 5, 6, 7, 3′, 4′-pentamethoxyflavone binds BCL2L1. **(E)** 5, 7, 5′-trimethoxy-3′, 4′-methylenedioxyflavone binds EIF2AK3. **(F)** 5, 6, 7, 5′-tetramethoxy-3′, 4′- methylenedioxyflavone binds EIF2AK3. **(G)** Molecular docking results between five PMFs and overlapping targets.

A total of 150 targets were predicted for the five PMFs by SwissTargetPrediction, while 229 targets related to the endoplasmic reticulum stress pathway were found in GeneCards. The Venn analysis of the “Disease-Drug-Pathway” interaction revealed that eight target genes, including EIF2AK3, APP, BCL2L1, AKT1, LRRK2, PIK3R1, ALOX5, and CFTR, shared among the gene sets associated with ALI, PMFs and ER stress (Figure 6A). This result suggest that these eight genes may serve as key therapeutic targets through which PMFs modulate the ER stress pathway to treat ALI. The results of molecular docking showed that the binding energies of the remaining seven targets to the five flavonoids were less than −1.2 kcal/mol, except for that of PIK3R1 (Figure 6G). Except for 5,6,7,3′,4′-pentamethoxyflavone, which had the lowest binding energy to the BCL2L1-encoded protein Bcl-XL (Figure 6D), the remaining four flavonoids had the lowest binding energies to the EIF2AK3 encoded protein PERK (Figures 6B–F), and the binding energy of PERK with 5, 7, 3′, 4′, 5′-pentamethoxyflavone reached - 5.62 kcal/mol (Figure 6C).

### 3.7 PMFs restore LPS-induced ER stress in the lung

**FIGURE 7:**
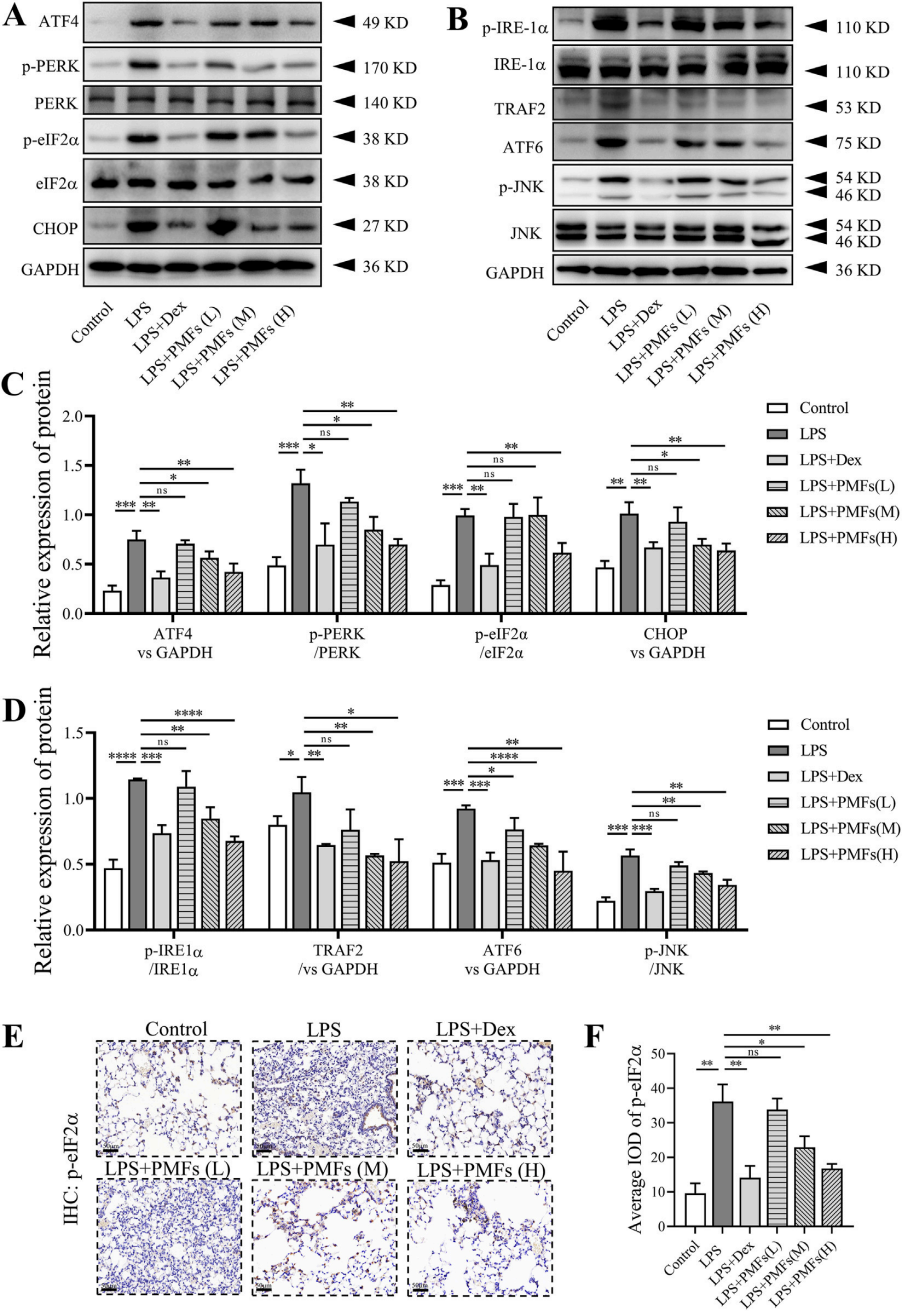
The effects of PMFs on LPS-induced ER stress. Data are presented as mean ± SD; n = 3, *p < 0.05, **p < 0.01, ***p < 0.001, ****p < 0.0001. **(A,B)** Western blot analysis of ER stress-relative protein including ATF4, PERK, p-PERK, eIF2α, p-eIF2α, CHOP, IRE-1α, p-IRE-1α, TRAF2, ATF6, JNK, p-JNK and GAPDH. **(C,D)** Quantitative analysis for ATF4, CHOP, TRAF2 and ATF6 expression as well as p-PERK/PERK, p-eIF2α/eIF2α, p-IRE-1α/IRE-1α and p-JNK/JNK ratio. **(E)** Representative immunohistochemistry with p-eIF2α antibody to lung tissue. Scale bar = 50 μm. **(F)** Quantification of the average IOD values for p-eIF2α.

To validate the prediction from molecular docking that PMF modulate LPS-induced ER stress, we investigated the levels of ER stress-related proteins in lung tissue. Western blot analysis showed that LPS activated different ER stress signaling pathways in the lungs, activating IRE1α, PERK, and ATF6, respectively. Treatment of mice with PMFs at doses of 300 and 900 mg/kg resulted in a significant reduction of ER stress signaling and a decrease in the level of eIF2α phosphorylation in the lungs of mice. PMFs blocked the JNK, CHOP, and TRAF2 apoptotic signaling pathways to alleviate lung cell injury (Figures 7A–D). Unexpectedly, PMFs at doses of 300 and 900 mg/kg also directly decreased the expression of p-eIF2α in lung tissues (Figures 7E,F), implying a potential role of PMFs in controlling ER stress.

### 3.8 Effects of PMFs on LPS-induced apoptosis in lung cells

**FIGURE 8:**
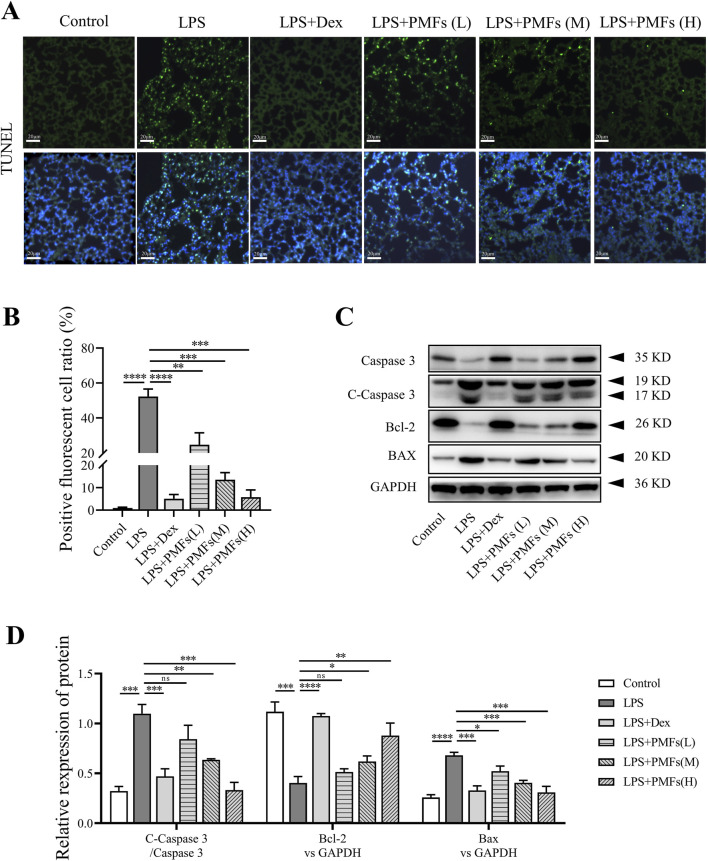
The effects of PMFs on LPS-induced lung cell apoptosis. Data are presented as mean ± SD; n = 3, *p < 0.05, **p < 0.01, ***p < 0.001, ****p < 0.0001. **(A)** Representative TUNEL staining in lung, the green dots are TUNEL stained positive cells. Scale bar = 20 μm. **(B)** Quantitative analysis for TUNEL positive cell ratio. **(C)** Western blot analysis of apoptosis-relative protein including Caspase 3, Cleaved-Caspase 3, Bcl-2, BAX and GAPDH. **(D)** Quantitative analysis for Bcl-2 and BAX expression as well as Cleaved-Caspase 3/Caspase 3 ratio.

To explore the mechanism by which PMFs antagonize LPS-induced ALI, we examined the progression of apoptosis in the lung. From the TUNEL results, it was evident that LPS induced apoptosis in lung cells by fracturing intracellular DNA to expose 3′-OH terminal to produce positive TUNEL results (Moore et al., 2021). PMFs have a significant role in protecting lung cells from LPS-induced apoptosis (Figures 8A,B). Treatment with PMFs obviously attenuated the number of apoptotic cells in mouse lung tissues, accompanied by a decrease in the expression of the pro-apoptotic protein BAX. LPS also decreased the expression of the anti-apoptotic protein Bcl-2 and accelerated the apoptotic process by increasing the ratio of cleaved caspase 3. PMFs effectively restored the expression level of Bcl-2 and reduced LPS-induced apoptosis by decreasing cleaved caspase 3 and BAX expression (Figures 8C,D). Dex, a hormonal anti-inflammatory agent, also exerted an anti-apoptotic effect on lung cells, suggesting that LPS-induced apoptosis in lung cells may be associated with inflammatory responses.

## 4 Discussion

Acute lung injury (ALI) is a predominant cause of death due to respiratory failure. It arises from direct insults, such as pneumonia and harmful inhalants, or indirect injuries including ischemia-reperfusion, aspiration of gastric contents, sepsis, multiple traumas, or acute pancreatitis. These events can lead to an unstable redox state, resulting in DNA damage, or oxidation of proteins and lipids (Mantell and Lee, 2000; Sarma and Ward, 2011). *Bauhinia championii* contains metabolites that have been investigated and verified to alleviate arthritis and counteract the LPS-induced inflammatory response (Liang et al., 2020; Xu et al., 2016). Nonetheless, the multitude of metabolites present within *B. championii* is diverse and complex, and the specific active metabolites beneficial for ALI treatment and their precise mechanisms remain to be elucidated. The network analysis indicated that the seven flavonoid metabolites in *B. championii* have potential therapeutic effects for ALI through involvement in the anti-inflammatory, antioxidant and anti-apoptotic pathways. Interestingly, five flavonoid constituents within PMFs extracted from *B. championii* were all encompassed among the seven metabolites predicted by network analysis. These computational findings suggest that PMFs possess a potential bioinformatic basis for treating acute lung injury (ALI).

LPS induces the release of severe pro-inflammatory factors and leads to the activation of inflammatory signaling in the lungs, creating a pneumonitis characterized by inflammatory leukocyte infiltration, which ultimately triggers apoptosis of alveolar cells or lung stromal cells, inducing lung dysfunction (Gong et al., 2021; Zhao et al., 2016). Our results show massive inflammatory cell infiltration in the lungs of mice on the second day after LPS administration, accompanied by elevated expression levels of inflammatory factors and activation of inflammatory signaling pathways that permeate the high oxidative stress signaling environment from the lung tissues into the trachea/bronchioles. According to modern pharmacology, polymethoxyflavonoids, which have anti-inflammatory and antioxidant effects, are often used in the treatment of neurological disorders, kidney diseases, and even (Chen et al., 2022). In the present study, we extracted a class of polymethoxyflavonoids, the main chemical substances of which were 5, 6, 7, 3′, 4′-pentamethoxyflavone, 5, 6, 7, 3′, 4′, 5′-hexamethoxyflavone, 5, 7, 3′, 4′, 5′-pentamethoxyflavone, 5, 6, 7, 5′-tetramethoxy-3′, 4′-methylenedioxyflavone and 5, 7, 5′-trimethoxy-3′, 4′-methylenedioxyflavone, with a total content of 90.18% in an extract from *B. championii*. It effectively alleviated LPS-induced ALI, significantly reduced inflammatory areas in mouse lungs, and restored lung tissue injury in a dose-dependent manner.

The pathogenesis of ALI involves a variety of mechanisms and is mainly exacerbated by oxidative stress and inflammatory signals in lung tissue (Luan et al., 2022). Previously, it was pointed out that oxidative stress manifesting as abnormal production of ROS and/or impaired antioxidant defenses can lead to cellular damage that triggers the accumulation of inflammatory cell-secreted pro-inflammatory cytokines such as TNF-α, IL-6, and IL-1β (Dhlamini et al., 2022). In LPS-induced ALI, TLR4 mediates oxidative stress signaling and promotes the NF-κB signaling pathway to induce inflammation, which disrupts the integrity of the alveolar-capillary barrier (Tang et al., 2021). Subsequently, inflammatory cells and protein-rich edema fluid are allowed to flow through the compromised barrier into the alveolar space (Johnson and Matthay, 2010). Similarly, our study demonstrated that PMFs alleviated LPS-induced accumulation of oxidative products, which were scavenged by PMF-activated HYP, CAT and GSH-Px. This is likely to be the mechanism of action of PMFs modulating the NF-κB signaling pathway to reduce the release of pro-inflammatory factors and decrease LPS-induced inflammatory responses (Feng et al., 2018).

LPS-induced pulmonary infection involves ER stress-mediated biological responses (Ku and Cheng, 2020). During this process, LPS stimulation promotes ER stress generation, leading to ROS accumulation in lung tissue. Huobao Yang et al. demonstrated that either suppressing Nrf2 or enhancing ER stress to amplify ROS production markedly reverses the anti-inflammatory effects conferred by CRBN knockout, thereby exacerbating ALI (Yang et al., 2020). In our study, the network analysis and *in vivo* pharmacodynamic evaluations collectively revealed the anti-inflammatory and antioxidant properties of PMFs. We hypothesize that PMFs ameliorate ALI-associated pulmonary pathology through ER stress regulation. By identifying shared targets among PMFs, ALI, and ER stress via molecular docking, this study revealed that PERK exhibits strong binding interactions with all five PMF constituents. These findings suggest that PMFs alleviate ALI by modulating ER stress, consistent with network-predicted mechanisms. The robust binding affinities between PERK and all five PMF-derived flavonoids, implying their potential to inhibit the PERK-eIF2α signaling axis, a key pathway in ER stress. This supports their capacity to suppress the PERK-eIF2α pathway by directly targeting PERK. Notably, LPS-induced ALI activates not only the PERK-eIF2α-mediated ER stress pathway but also IRE1-and ATF6-driven ER stress responses (Pang et al., 2020). Our experimental data corroborated these findings: LPS stimulation significantly increased phosphorylated PERK, phosphorylated IRE-1α, and ATF6 upregulation in murine lung tissues, confirming ER stress induction. Conversely, PMFs exerted negative regulation across the LPS-activated ER stress cascade.

Under ER stress, cell death is generally considered as the ultimate direction of mitochondria-associated apoptosis by an overload of unfolded or misfolded proteins in response to cell stress (Gong et al., 2017). This type of cell death is often attributed to the inflammatory response and the accumulation of intracellular ROS in ALI (Li et al., 2022; Sang et al., 2022). As a key effector of ER stress, eIF2α is activated upon receiving signals from unfolded proteins, and this subsequently leads to PERK activation and contributes to apoptosis. This process represents a detrimental response in lung tissue (Li et al., 2023; Wang et al., 2022). As shown in our results, LPS activated PERK, phosphorylates eIF2α, increased ATF4 and CHOP expressions and increased ER stress. PMFs downregulated both PERK and IRE1α phosphorylation, decreased ATF4 and CHOP expression, and blocked NF-κB inflammatory signaling and JNK apoptotic signaling, inhibiting apoptosis. This apoptotic signaling promoted Caspase 3-mediated activation of the mitochondrial apoptotic pathway, eliciting cell death in lung tissue and ultimately provoking acute lung injury.

## 5 Conclusion

**FIGURE 9:**
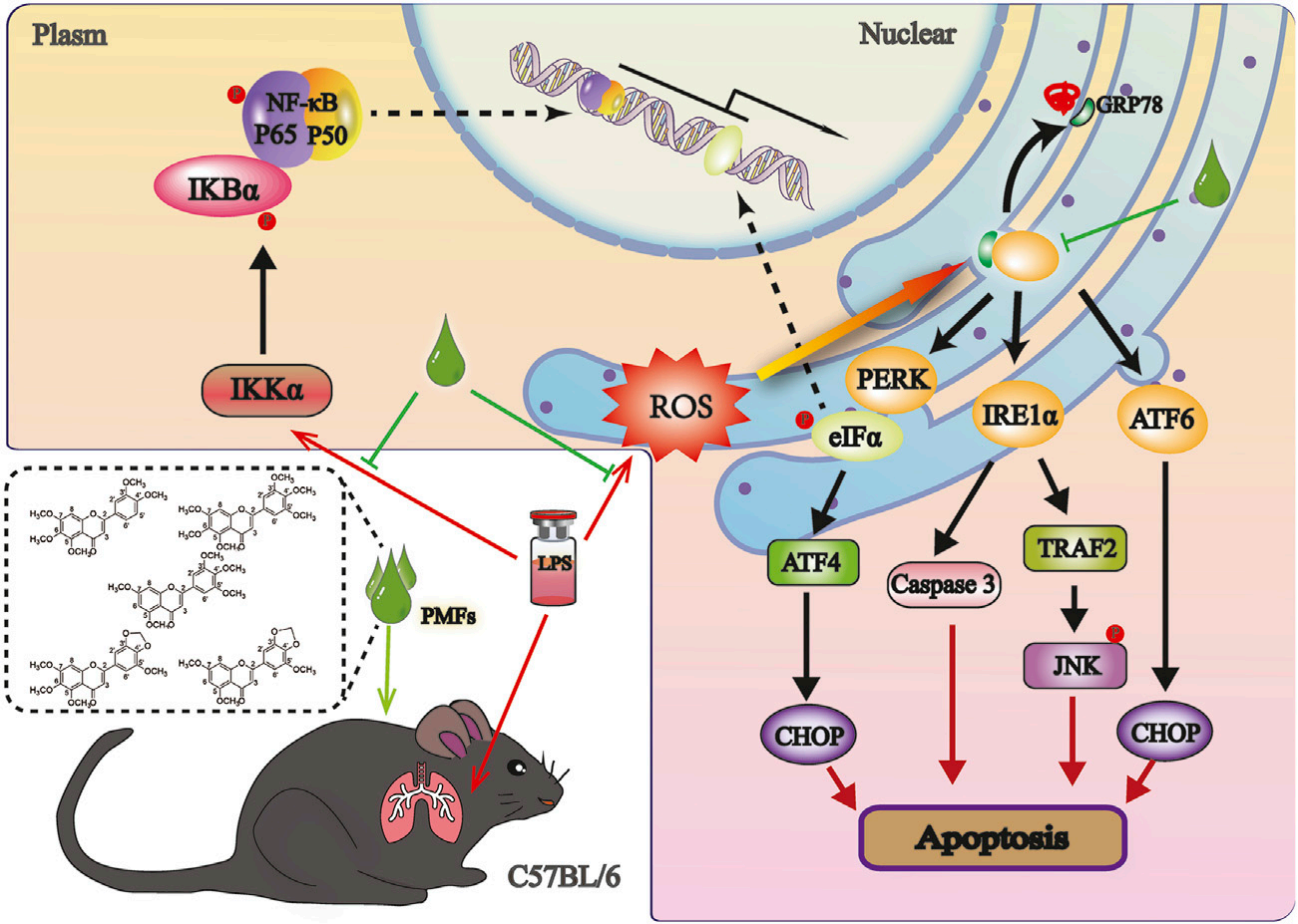
A Schematic representation of the mechanism underlying for PMFs to antagonize LPS induced ALI. LPS induced a ROS outbreak and upregulated transcription of proinflammatory factors, those augment UPR, thereby promoting ER stress and ultimately apoptosis. PMFs treatment reduced the production of ROS and pro-inflammatory cytokines for releasing ER stress to protect lung cells in mice.

In the present study, a class of polymethoxyflavonoids, PMFs, was extracted and isolated from *B. championii*. The results of network analysis indicated that PMFs possessed anti-inflammatory, antioxidant and anti-apoptotic effects, which were confirmed by *in vivo* experiments. In addition, molecular docking and signaling pathway validation also indicated that PMFs have the potential to act on ER stress in lung tissue cells, thereby alleviating acute lung injury (Figure 9). These results provide a valid preclinical research basis for the clinical use of PMFs.

## Data Availability

The original contributions presented in the study are included in the article/Supplementary Material, further inquiries can be directed to the corresponding author.
