# Supplementary material for: Polymethoxyflavones extracted from Bauhinia championii alleviate LPS-induced acute lung injury by ameliorating endoplasmic reticulum stress
Source: Front Pharmacol. 2025 Jun 11;16:1544916. doi: 10.3389/fphar.2025.1544916 (PMC12187725; doi:10.3389/fphar.2025.1544916)
Supplement: Supplementary file 2 [file DataSheet1.docx]

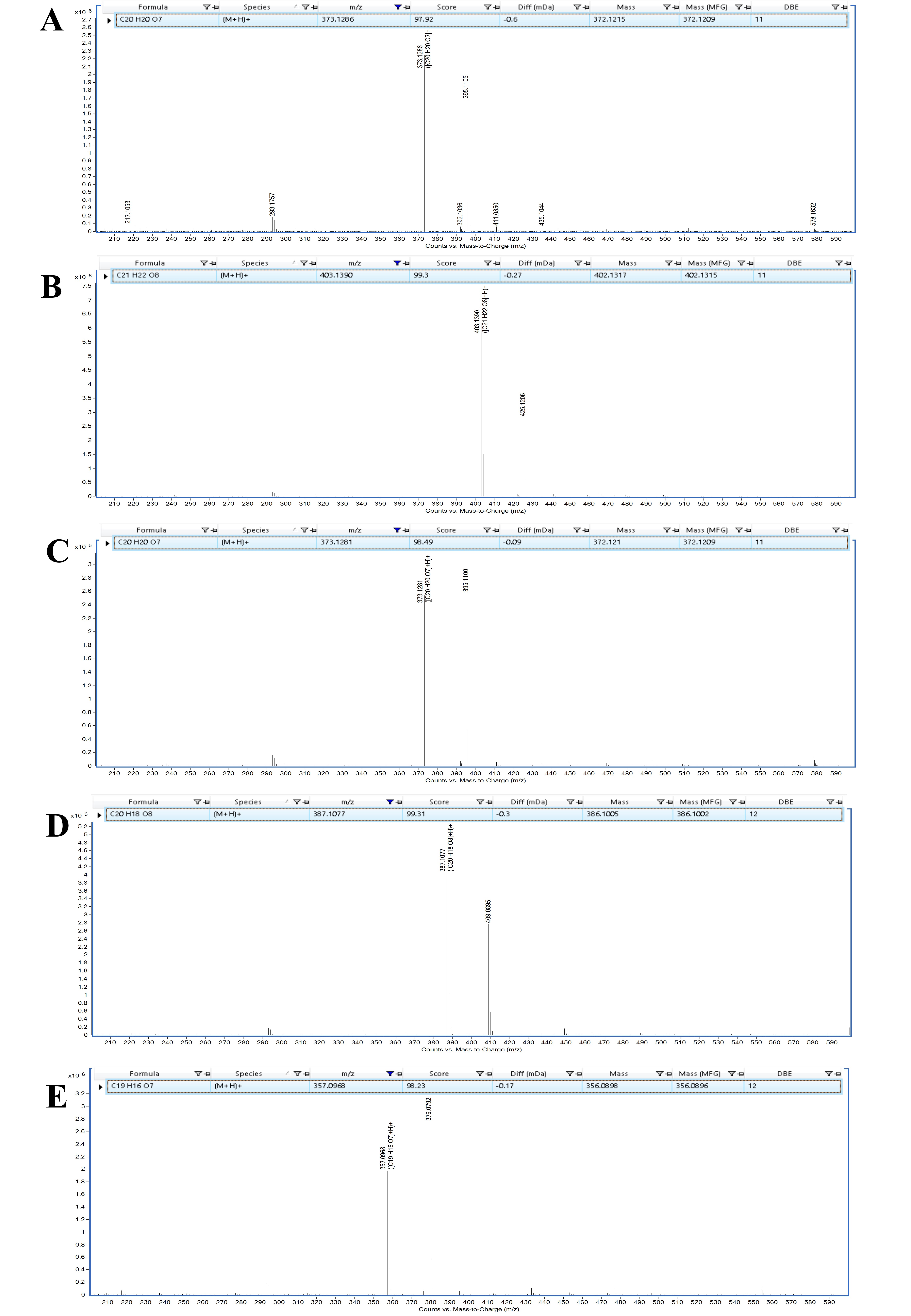


**Supplemental Figure. 1** The high-resolution mass spectrometry data and mass spectra of PMF1-5. **A** 5,6,7,3',4'-pentamethoxyflavone (PMF 1). **B** 5,6,7,3',4',5'-hexamethoxyflavone (PMF 2). **C** 5,7,3',4',5'-pentamethoxyflavone (PMF 3). **D** 5,6,7,5'-tetramethoxy-3',4'-methylenedioxyflavone (PMF 4). **E** 5,7,5'-trimethoxy-3',4'-methylenedioxyflavone (PMF 5).

**Supplemental Figure. 2**. The MS^2^ spectrum (precursor-ion was 373([M+H]^+^))of PMF 1.


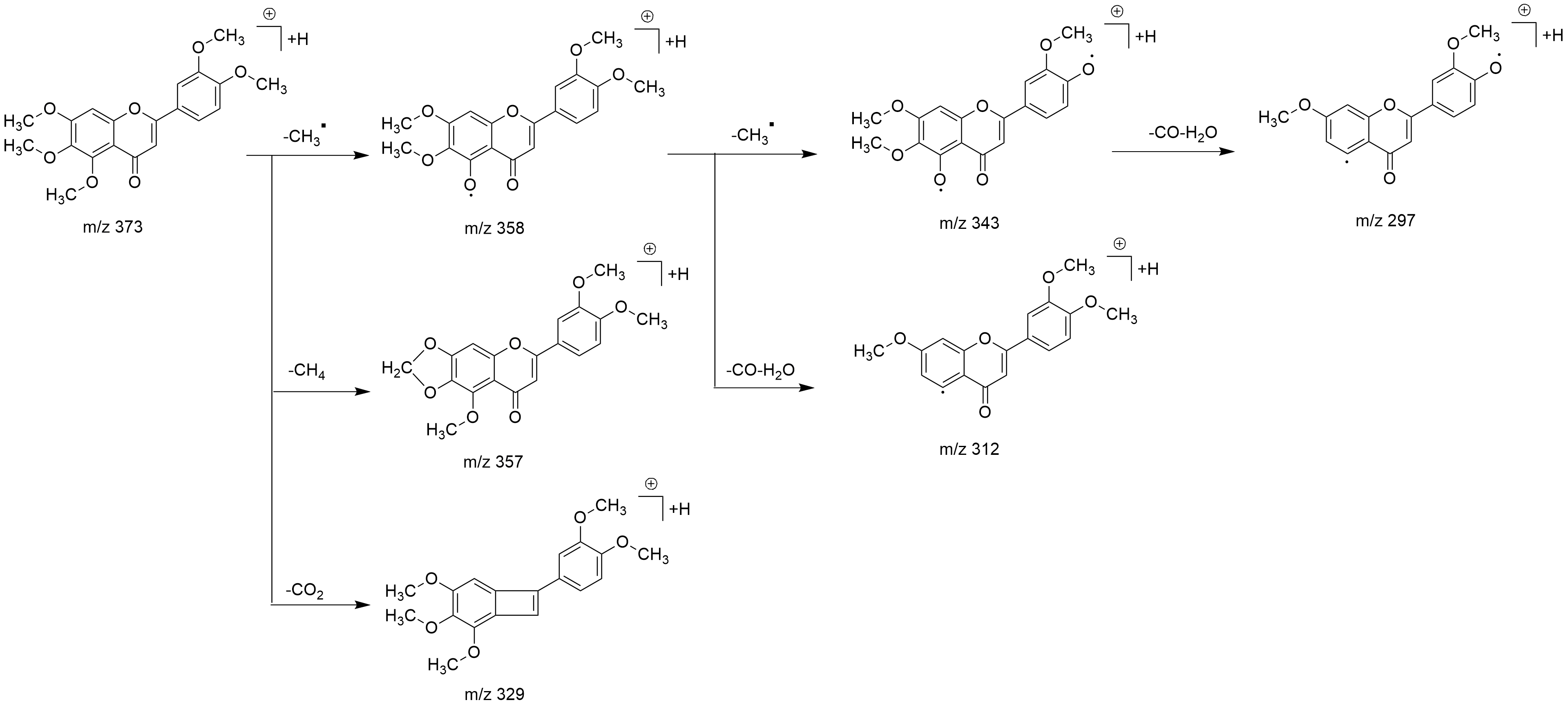


**Supplemental Figure. 3**. Proposed fragmentation pathway of PMF 1 in ESI positive mode.

**Supplemental Figure. 4**. The MS^2^ spectrum (precursor-ion was 403([M+H]^+^))of PMF 2.


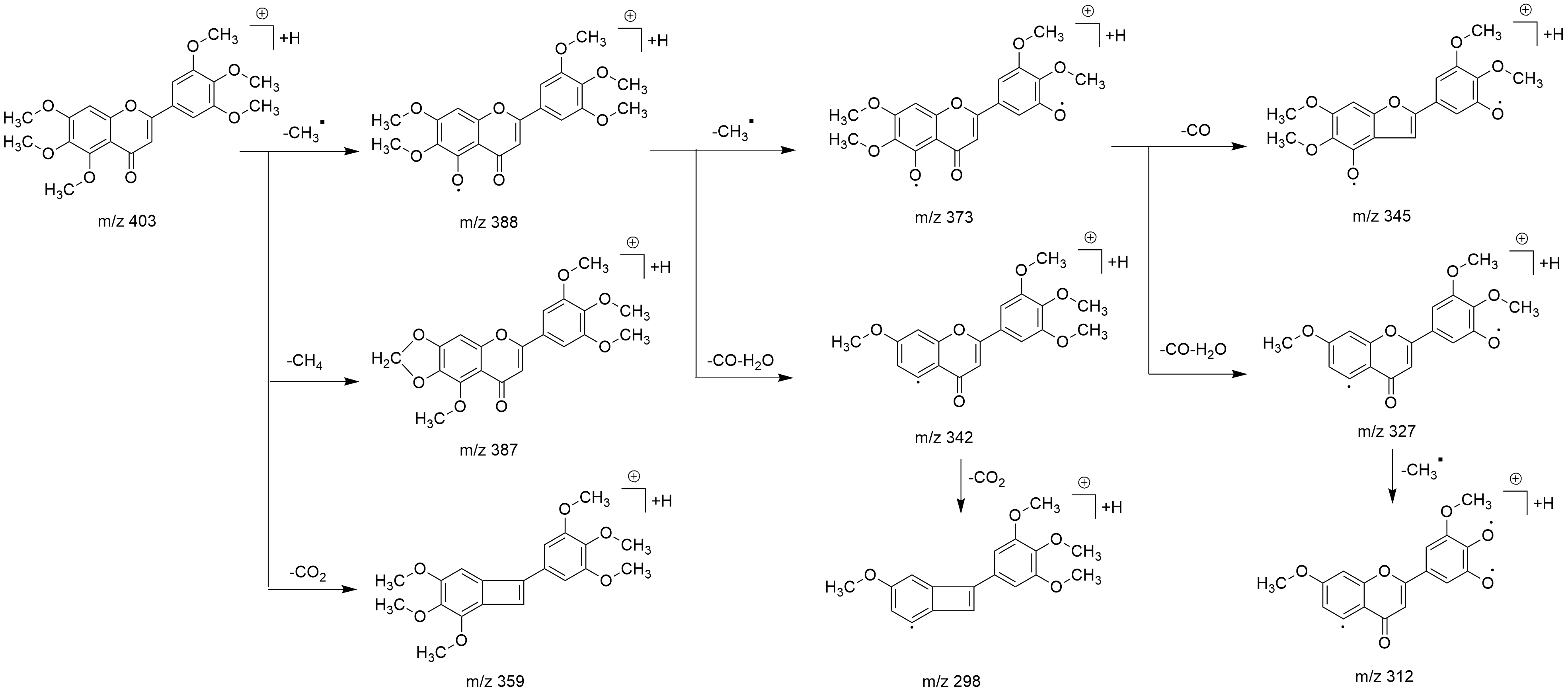


**Supplemental Figure. 5**. Proposed fragmentation pathway of PMF 2 in ESI positive mode.

**Supplemental Figure. 6**. The MS^2^ spectrum (precursor-ion was 373([M+H]^+^))of PMF 3.


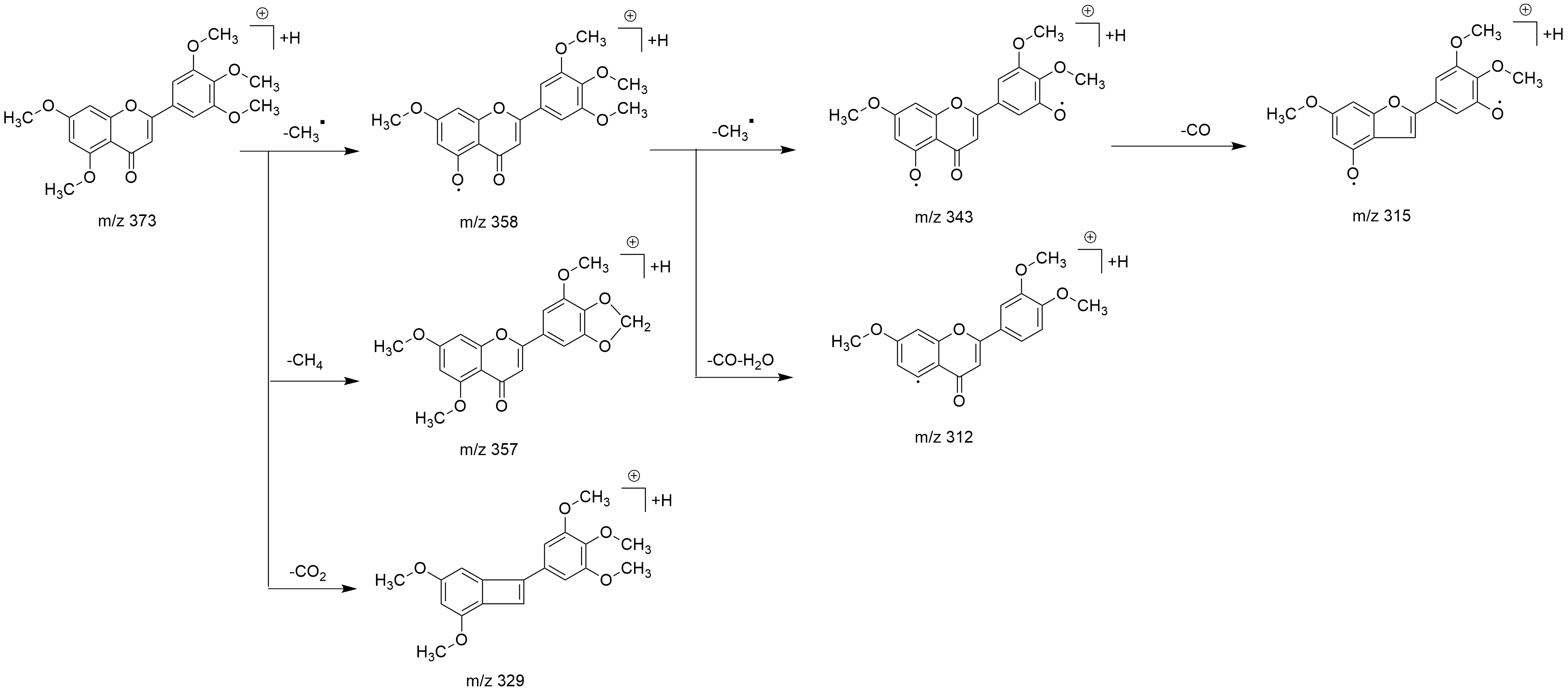


**Supplemental Figure. 7**. Proposed fragmentation pathway of PMF 3 in ESI positive mode.

**Supplemental Figure. 8**. The MS^2^ spectrum (precursor-ion was 387([M+H]^+^))of PMF 4.


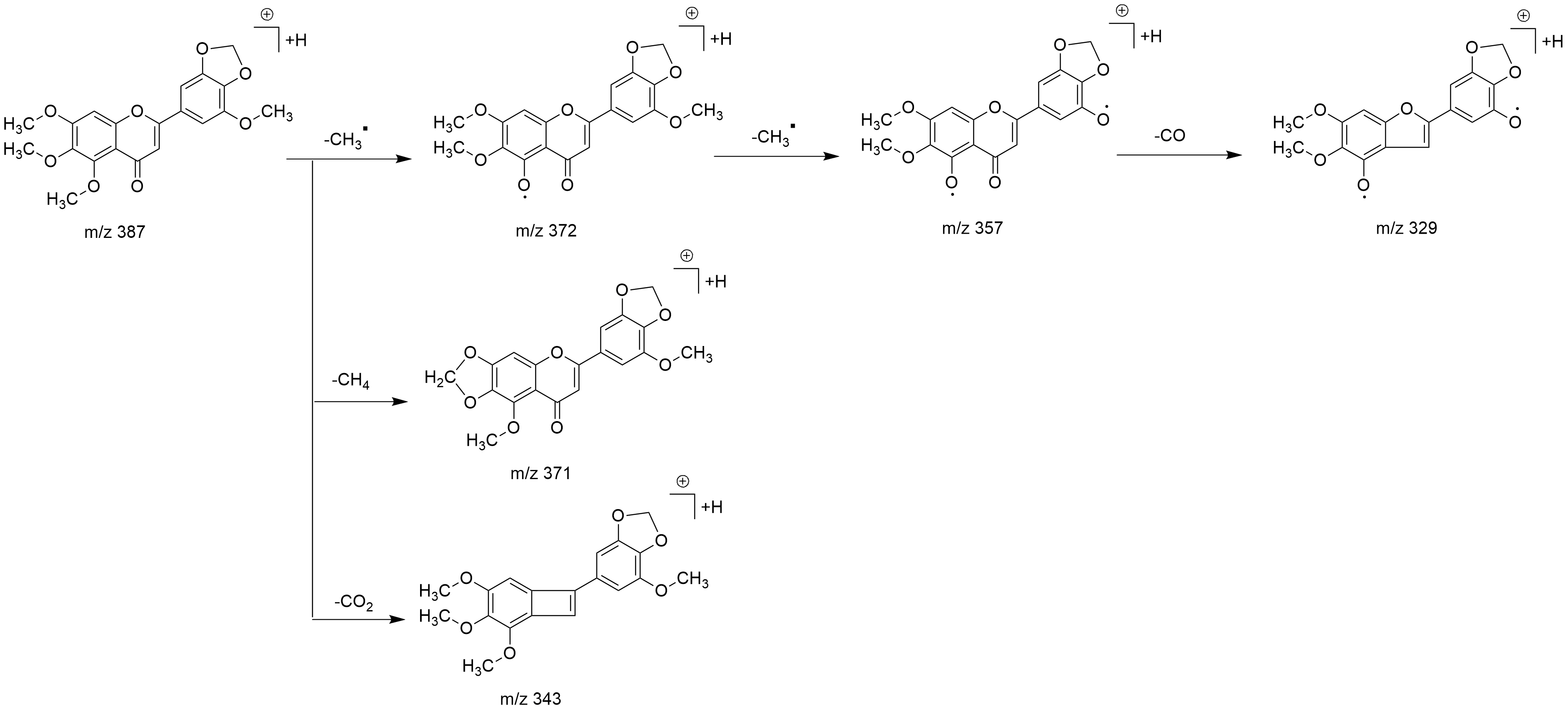


**Supplemental Figure. 9**. Proposed fragmentation pathway of PMF 4 in ESI positive mode.

**Supplemental Figure. 10**. The MS^2^ spectrum (precursor-ion was 357([M+H]^+^))of PMF 5.


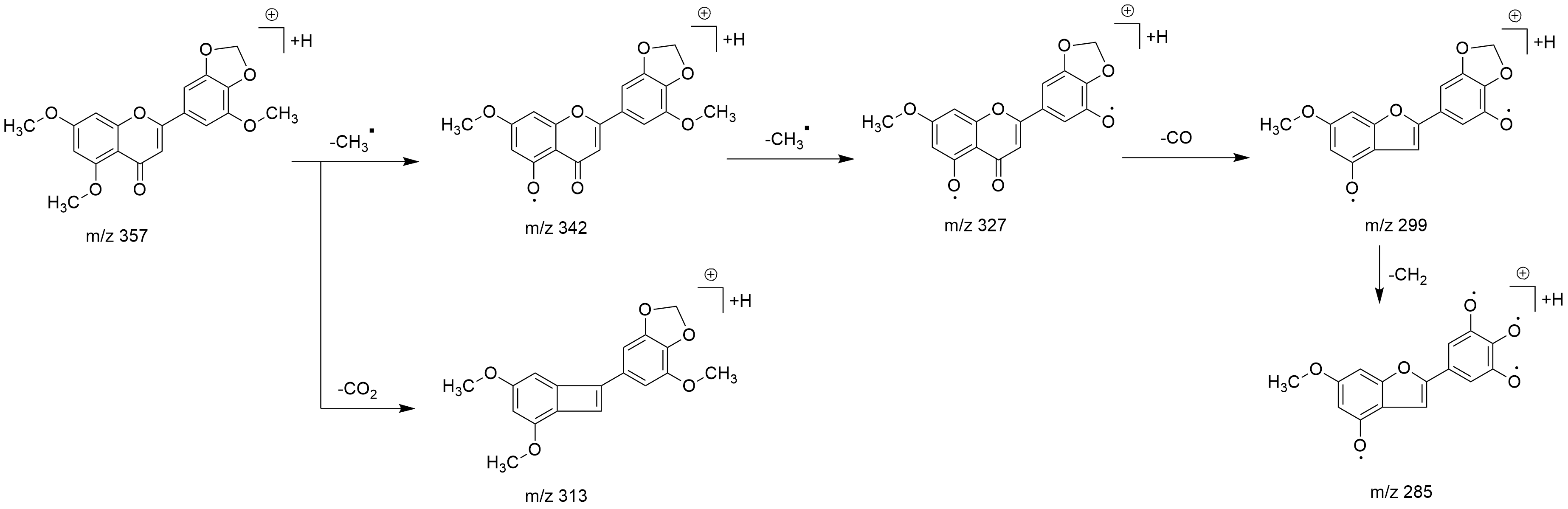


**Supplemental Figure. 11**. Proposed fragmentation pathway of PMF 5 in ESI positive mode.
